# Skin Rejuvenation with Non-Invasive Pulsed Electric Fields

**DOI:** 10.1038/srep10187

**Published:** 2015-05-12

**Authors:** Alexander Golberg, Saiqa Khan, Vasily Belov, Kyle P. Quinn, Hassan Albadawi, G. Felix Broelsch, Michael T. Watkins, Irene Georgakoudi, Mikhail Papisov, Martin C. Mihm Jr., William G. Austen Jr., Martin L. Yarmush

**Affiliations:** 1 Center for Engineering in Medicine, Department of Surgery, Massachusetts General Hospital, Harvard Medical School, and Shriners Burns Hospital, Boston, MA, 02114; 2 Porter School of Environmental Studies, Tel Aviv University, Tel Aviv, Israel; 3 Division of Plastic and Reconstruction Surgery, Massachusetts General Hospital, Harvard Medical School, Boston, MA 02114; 4 Department of Radiology, Massachusetts General Hospital, Harvard Medical School, and the Shriners Burns Hospital, Boston, MA, 02114; 5 Department of Biomedical Engineering, Tufts University, Medford, MA, 02155; 6 Division of Vascular and Endovascular Surgery, Massachusetts General Hospital, Harvard Medical School, Boston, MA 02114; 7 Department of Dermatology, Brigham and Women’s Hospital, Harvard Medical School, Boston, MA 02115; 8 Department of Biomedical Engineering, Rutgers University, Piscataway, NJ 08854

## Abstract

Degenerative skin diseases affect one third of individuals over the age of sixty. Current therapies use various physical and chemical methods to rejuvenate skin; but since the therapies affect many tissue components including cells and extracellular matrix, they may also induce significant side effects, such as scarring. Here we report on a new, non-invasive, non-thermal technique to rejuvenate skin with pulsed electric fields. The fields destroy cells while simultaneously completely preserving the extracellular matrix architecture and releasing multiple growth factors locally that induce new cells and tissue growth. We have identified the specific pulsed electric field parameters in rats that lead to prominent proliferation of the epidermis, formation of microvasculature, and secretion of new collagen at treated areas without scarring. Our results suggest that pulsed electric fields can improve skin function and thus can potentially serve as a novel non-invasive skin therapy for multiple degenerative skin diseases.

Skin is the largest organ in the body and has important functional and psychological significance. Aging, trauma, and chronic metabolic diseases, including diabetes, often lead to alterations in skin color, texture, and barrier function. Loss of skin function leads to atrophy, infection, chronic wounds, laxity, and rhytides^1^. Epidermal atrophy – characterized by a thinning of the epidermis and an increase in fragility – is observed in ~32% of individuals over 60 years of age^2^. Thus, it is not surprising that 2,156,075 skin rejuvenation procedures were performed in 2013 in the United States alone^3^.

Because these skin diseases are often associated with poor re-epithelialization, poor blood supply, reduced collagenesis and a loss of collagen functional properties, current rejuvenation therapies are focused on the removal of nonfunctional tissue and the induction of de novo growth of healthy dermal cells, blood vessels, and extracellular matrix. Common methods to induce proliferation of the epidermis and angiogenesis include topical application of silicone mesh, antiseptic gels, and growth factors^4^, *in vitro* cultivated cellular sheets^5^, gene therapy^6^, ultraviolet light^7^, electrical stimulation^7^, ultrasound therapies^7^, extracorporeal shock wave therapy^8^, and low intensity vibrations^9^. Therapies that aim to induce collagen synthesis and to restore skin function and appearance include non-ablative laser technologies, radiofrequency, ultrasound, electro-optical synergy, chemical peels, microdermabrasion, injectable fillers, neurotoxins, skin needling, mesotherapy, platelet-rich plasma, and cell therapies^10^. However, physical and chemical methods of the listed therapies have certain disadvantages. The major disadvantage of current physical rejuvenation methods is that they deliver external energy to the whole tissue bulk, affecting both cells and extracellular matrix; this changes the function and architecture of treated tissue. The major disadvantage of chemical rejuvenation therapies is that even though they target only cells, they involve the delivery of external molecules that can cause an off-target tissue response. This uncontrollable subsequent reaction might result in clinical complications such as burns, skin vascular malformation, tumors^11^, keloids, hypertrophic scarring, skin contraction, paralysis of facial muscles, necrosis, intravascular penetration, and infection^12^,^13^.

The goal of this paper is to introduce a new non-invasive method for induction of skin rejuvenation using pulsed electric fields (PEF). Unlike contemporary physical methods that affect all tissue components, PEF is an intervention at the cellular level, which precisely targets cell membranes through electroporation without affecting the extracellular matrix architecture^14^. Unlike chemical interventions, PEF is a non-invasive procedure that does not involve the application of external molecules. In relation to skin therapies, PEF based technologies are under clinical investigation for small molecule and gene delivery, DNA vaccination, and tumor ablation^15^,^16^. In a previous study, we have also demonstrated, using a rat model, that PEF-ablated skin regenerates without scars^17^. Here we report that certain PEF regimens applied non-invasively lead to epithelial growth, an increase in vascular supply to the skin, the formation of new microvasculature, and increased collagen deposition without scarring. In addition, we describe a set of numerical models that simulate electric field distribution in different layers of normal rat skin. These numerical tools can be used for clinical PEF treatment planning and elucidation of the fundamental effects of various PEF regimens on dermal cells. Our results suggest that PEF can serve as a novel therapy for controlled skin rejuvenation.

## Results

### Optimization of PEF parameters: voltage, pulse duration, and number of pulses for induction of skin collagenesis

**Figure 1:**
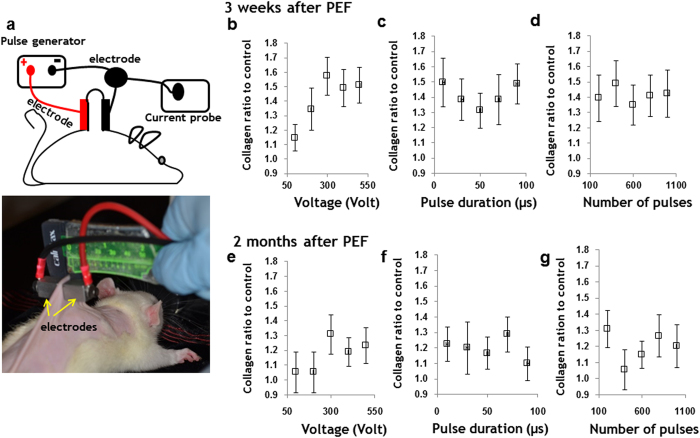
PEF system and protocol optimization. **a** Schematic representation of the experimental setup and digital image of electrodes used for PEF administration. **Individual impact of PEF parameters on collagenesis. Three weeks after treatment (a-c) two months after treatment (d-f)**. The effect of each individual parameter was calculated using Taguchi method. **a** and **d** show the effect of voltage increase on the new collagen synthesis three weeks and two months after PEF administration. **b** and **e** show the effect of increase of pulse duration on the new collagen synthesis at three weeks and two months after PEF. **d** and **f** show the effect of increase of number of pulses on the new collagen synthesis three weeks and two months after PEF administration. Three animals were used for each time point and each parameter. Four animals were used as a control. Error bar shows ± SEM.

Pulsed electric field experimental protocol includes multiple possible parameters: 1) electric field strength (*E*, Vmm^−1^), 2) pulse duration (*t*_*p*_, μs), and 3) number of pulses (*N*). The choice of these parameters often depends on the experience of the researcher regarding the specific electroporation system; therefore, multiple parameters appear in the literature and lead to significant confusion. Moreover, since these parameters need to be applied simultaneously during the PEF treatment, the individual role of each of the parameters in the process is unclear. Hence, the goal in this first series of experiments was to determine the effects of pulsed electric field parameters on skin collagenesis, as controlled collagenesis differentiates skin rejuvenation from scarring. The range of PEF parameters and their combinations is large. Therefore, to decrease the number of experiments but still delineate the impact of each parameter independently, we applied the Taguchi Orthogonal Array to the experimental design^18^. We tested the impact of applied voltage in the 100–500 V range- which determines the local *E* as modeled in Fig. S1- N (200-1000 range) and *t*_*p*_ (10–90 μs range). For PEF delivery, two electrodes were applied on the skin flap as shown in Fig. 1a. Supplementary information 1 describes the Taguchi analysis performed to determine the significance (rank) of each PEF component independently. Following the Taguchi methodology, we tested 5 doses (levels) of each of the parameters. The experimentally tested values are shown in Table S1. The experimental results of the impact of the tested PEF protocols (Table S1) on collagen synthesis in rat skin are shown in Table S1. The experiments for the L25 orthogonal Taguchi array and Taguchi Signal to Noise (S/N) ratio calculated for each of the conducted experiments are summarized in Table S2. The efficacy range for each of the factors was calculated using Eq. S7 (Supplementary information 1). Highest ranking -1- was assigned to the parameters with the largest effect.

The individual effects of each of the tested factors on collagen synthesis three weeks after the single PEF treatment of normal rat skin are summarized in Table 1. As it can be seen, the parameter of pulse duration has the most prominent impact on collagen synthesis (Taguchi Rank 1). Increasing the pulse duration generally increases the collagen content in the skin. Applied voltage is ranked second (Taguchi Rank 2) in significance on collagen level elevation, whereas number of pulses has the smallest effect on increasing collagen level elevation (Taguchi Rank 3).

The individual responses for each of the tested factors on collagen synthesis two months after a single PEF treatment of normal rat skin are shown in Table 2. As shown, the number of pulses shows the most prominent effect on the maintenance of increased collagen content in the skin (Taguchi Rank 1). Applied voltage and pulse duration have similar effects (equal Taguchi ranks).

The normalized individual effect of applied voltage, pulse duration, and number of pulses on total collagen content increase in normal rat skin, measured by a hydroxyproline assay are shown in Fig. 1. The impact of the applied voltage on total collagen content in the skin is shown in Fig. 1b,e; and the respective distribution of *E* (Vmm^−1^) in various skin layers at the applied voltages appears in Fig. S1c. For control we used a normal, untreated skin from age matched animals.

Application of 100 V increased the collagen content ratio by 1.15 ± 0.09 in comparison to untreated skin, three weeks after PEF administration. Two months after treatment, the collagen concentration returned to basal levels. Application of 200 V increased the collagen content ratio by 1.34 ± 0.14 as compared to untreated skin, three weeks after PEF administration. Two months after treatment, the collagen concentration returned to basal levels. Increasing the applied voltage to 300 V, elevated the total collagen content ratio by 1.57 ± 0.13 in comparison with untreated skin, three weeks after PEF administration. Two months after treatment, the collagen content in the skin was still greater than basal levels by a factor of 1.31 ± 0.13. Further increase of voltage to 400 V and 500 V did not cause an additional increase in the collagen content ratio in comparison with untreated skin—neither three weeks nor two months after PEF administration.

The impact of pulse duration on collagenesis in skin is shown in Fig. 1c,f. Increasing the pulse duration from 10 μs to 90 μs causes an elevated collagen content by 1.41 ± 0.01 as compared to basal levels three weeks after PEF administration, and by 1.20 ± 0.12 two months after PEF administration. However, we did not find a correlation between pulse duration and the level of collagen synthesis.

The impact of number of pulses on the collagen content in skin is shown in Fig. 1d,g. Increasing the number of pulses from 200 to 1000 elevated the collagen content by 1.41 ± 0.14 in comparison with basal levels three weeks after PEF administration, and by 1.20 ± 0.12 two months after PEF administration. However, we did not find a correlation between the number of pulses and the level of collagen synthesis.

Based on the results from the Taguchi studies, we composed a PEF protocol to more fully examine the potential of PEF on skin rejuvenation. Applied voltage: 500 V (caused to significant elevation in collagen content, Fig. 1b,e). Pulse duration: 70 μs (led to significant elevation in collagen content Fig.1c,f). Number of pulses: 200 (led to significant elevation in collagen content Fig. 1d,g). Frequency of pulses delivery: 3 Hz (the same frequency was used in all experiments; the optimization of pulse delivery frequency is the subject of our future research).

The average measured current during PEF administration using this protocol was 2.21 ± 0.18 Amp (n = 41). Importantly, the tissue conductivity and thus the current change during application of electric fields^19^, and here we report the average values of the current measured in the experiment after each pulse. Skin rejuvenation was assessed through the quantification of 1) proliferation of the epidermis, 2) increase in collagen fiber density, and 3) angiogenesis.

### Non-thermal pulsed electric fields induce rat skin epidermis proliferation

**Figure 2:**
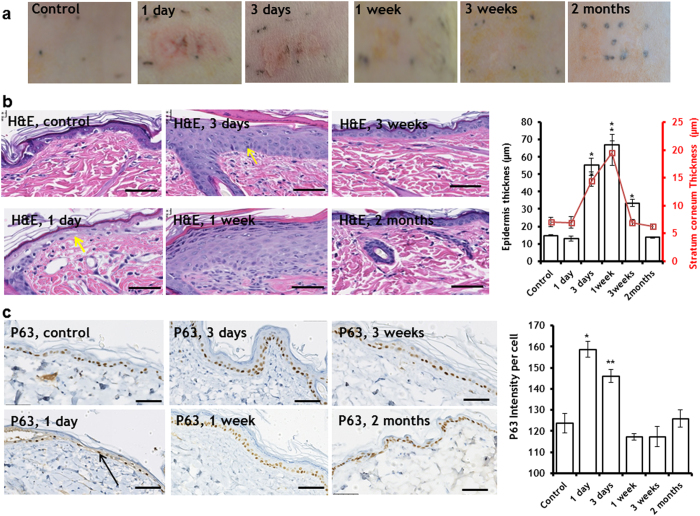
Induction of skin epidermis proliferation with PEF. **a** Digital photos of the PEF treated rat skin two months after treatment. **b** Dynamics of thickened skin epidermis and resolution to the baseline levels. Images show H&E staining of the PEF-treated epidermis. The plots show the average thickness of the epidermis and stratum corneum. (*) p-val < 0.001; **c.** The impact of PEF on p63 expression levels in the epidermal keratinocytes. Images show the immunohistochemical staining of p63 at various time points up to 2 months after PEF administration. The plot on the right shows the average level of p63 staining intensity in the epidermal keratinocytes. (*) p-val < 0.001; (**) p-val = 0.0011. Five animals per time point, five measurements per animal. Scale bar 50 μm. Error bar shows ± SEM. Statistical analysis was performed by first using one-way ANOVA for multivariate analysis and post hoc using Dunnett’s tests to assess significance between individual groups. Significance was set at *P* < 0.05.

PEF treated areas (1 cm^2^) of dorsal rat skin were monitored for up to two months after PEF administration. Digital photographs of the PEF treated skin appear in Fig. 2a. The treated area was marked with permanent black tattoo ink. One day after PEF administration, erythema at the treated skin site is observed. Three days after PEF administration, the redness was still appreciated at the treated areas. One week after PEF administration, the redness resolved, and the color of the PEF treated areas was paler than the surrounding skin. The texture of the PEF-treated site was also smoother than the surrounding untreated areas. Three weeks after treatment, the PEF-treated skin had a paler color than the surrounding tissue. In some animals, orange-tinged pigmentation appeared at the edge of the treated sites. Two months after PEF administration, the PEF treated areas could not be visually distinguished from the surrounding untreated skin.

Regarding histological examination, one day after PEF administration, the epidermis showed areas of focal necrosis overlaying new regenerative epithelium (basal cells and some squamous cells), with the result of an inter epidermal cleft (Fig. 2b, Fig.S3). Prominent subcorneal and intra epidermial abscesses were observed (Fig.S3). The abscesses mark the dead, not yet cleaned tissue beneath the preserved stratum corneum with a marked boarder line defined by neutrophils with actively proliferating epidermis beneath. The basement membrane of the epidermis was preserved throughout the whole area of the treated skin. Three days after PEF administration, rat skin exhibited hyperkeratosis with increased thickening of the epidermis (55.50 μm ± 3.95 μm in the PEF treated skin vs. 14.78 μm ± 0.53 μm in the untreated skin) as well as occasional apoptotic basal cells (Fig. 2b, yellow arrow). Three weeks after PEF administration, the stratum corneum was normal and the number of cell layers in the epidermis was reduced, though the epidermis was still thicker in the PEF treated areas as compared to untreated skin (33.48 μm ± 1.94 μm in the PEF treated skin vs 14.78 μm ± 0.53 μm in the control), (Fig. 2b). Two months after PEF administration, both the stratum corneum and the epidermis returned to their baseline thicknesses, similar to untreated skin (13.74 μm ± 0.44 μm in the PEF treated skin vs 14.78 μm ± 0.53 μm in the untreated skin), (Fig. 2b).

To investigate the mechanism of epidermal proliferation, we studied the expression levels of p63, which regulates proliferation and differentiation of mature keratinocytes and is a marker for keratinocyte stem cells^20^,^21^. A striking overexpression of p63 was observed in the keratinocytes present in the epidermis one day after PEF administration (28 ± 5% increase over control, Fig. 2c). Cells that show a prominently dark staining also were increased in size and had the appearance of cells migrating through the basement membrane (Fig. 2c, black arrow). This overexpression was still present three days after PEF administration (18 ± 5% increase over control). The expression of p63 in the epidermis returned to basal levels one week after PEF application. In addition to p63 expression levels, we quantified the release of growth factors and cytokines at the PEF-treated area (Fig.S1). We detected significantly increased levels of the following factors which were previously shown to enhance keratinocyte proliferation and re-epithelialization: IL-6^22^, EGF^22^, and VEGF^23^ (Fig.S4).

### The impact of PEF on dermal collagen fiber synthesis, orientation, density and mechanical properties

**Figure 3:**
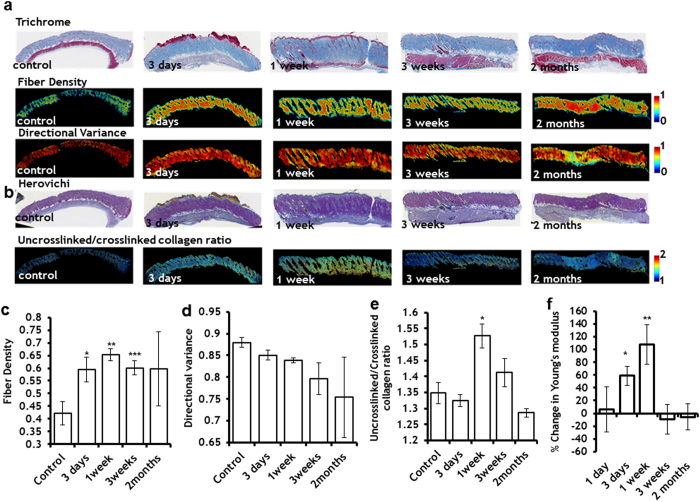
The effect of PEF on the extracellular matrix. **a** Top ( Maisson’s Trichrome staining) enabled the identification of collagen-containing pixels and demonstrated significantly higher density within the PEF-treated areas of the skin. Lower panel shows the pixel-wise fiber directional analysis, enabled quantification of the local strength of collagen fiber alignment, which demonstrated reduced variance in fibers up to three weeks after PEF, but no significant difference in the directional variation within the PEF treated area two months after the treatment. **b** Secretion of new fibers to the PEF-treated area was detected by Herovici staining. **c** Quantitative analyses of fiber density in the center of PEF treated area were significantly elevated, starting with day 3 after PEF treatment and continuing up to 3 weeks after the treatment. Two months after treatment the fiber density of the PEF treated area was not significantly different from the control, untreated skin (*) p-val = 0.03; (**) p-val = 0.009; (***) p-val = 0.02. **d** Quantitative analyses showed no significant difference in fiber alignment, indicating no scar formation at the PEF-area. **e** Quantification of blue/red in the digitized Herovici stained slides shows significant increase of the blue staining (collagen type III) one week after the PEF. (*) p-val = 0.01. For each experimental point we analyzed at least 4 slides, each obtained from a different animal. **f** Young’s modulus change of the PEF treated skin in comparison to untreated control: An increase in Young’s modulus was observed 3 days and 1 week after PEF treatment of the skin.(*) p-val = 0.0048 (**) p-val = 0.0028 Five animals were used for each time point. In all plots, error bars show ± SEM. Statistical analysis was performed by first using one-way ANOVA for multivariate analysis and post hoc using Dunnett’s tests to assess significance between individual groups. Significance was set at *P* < 0.05.

To further test the rejuvenation properties of PEF on the skin, we tested the impact of 200 pulses on dermal extracellular matrix, delivered at 500 V, 70 μs duration, and 3 Hz. Importantly, no scar formation was observed up to two months after PEF administration. Using previously developed image processing algorithms^24^, we quantified the fiber density and orientation in the histological sections. Figure 3a shows the Masson’s Trichrome staining of the PEF treated skin at different time points after the PEF treatment, which color maps of fiber density and fiber directional variance are shown in Fig. 3c,d. An increase in the fiber density in the center of the PEF treated skin is clearly observed up to 1 week after the PEF administration (55 ± 17% increase), followed by a reduction three weeks after PEF treatment by 8 ± 5% in comparison to maximum increase (Fig. 3a,c,d). Two months after PEF administration, the fiber density was not significantly different from that of control tissue. Moreover, the fiber directional variance was not significantly different from untreated skin at all tested time points, indicating that the process did not lead to increased fiber alignment that is indicative of scar formation.

Next, using a Herovichi stain, we detected the appearance of new, uncrosslinked collagen fibers (blue stain indicates uncrosslinked collagen III, while the red stain indicates crosslinked mature collagen type I^25^) (Fig. 3b,e). The maximum increase in collagen III (13 ± 3%) was detected one week after PEF administration. The level of uncrosslinked collagen gradually decreased over time (most likely due to the maturation of new fibers), reaching basal levels two months after treatment (Fig. 3e).

Finally, we tested the mechanical properties of the skin treated by PEF. The largest increase in the Young’s modulus (107 ± 13%) was observed one week after PEF administration (Fig. 3f) and it was correlated with the maximum deposition of new, uncrosslinked collagen III in the PEF treated skin (Fig. 3e). Three weeks after PEF administration, the Young’s modulus in the PEF treated skin was not different from the untreated skin.

### Non-thermal pulsed electric fields catalyze skin angiogenesis

**Figure 4:**
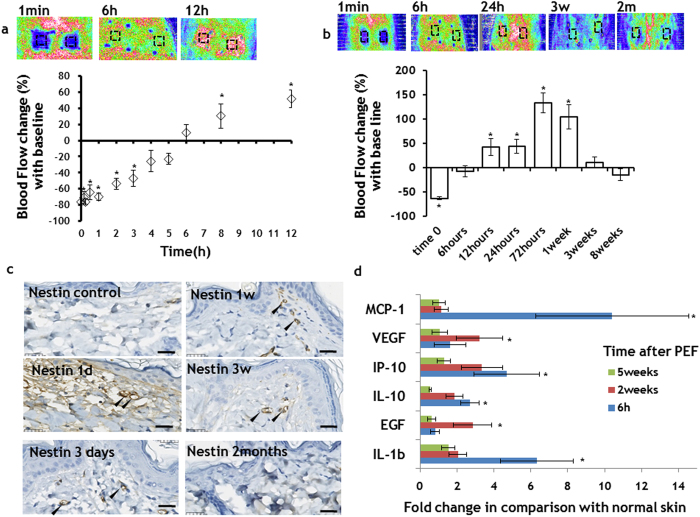
Controllable angiogenesis induction in the rat skin with PEF. **a** PEF led to a temporary vasoconstriction at the treated site. The flow returned to the baseline levels 5 hours after PEF application. In the following hours, we observed increase of the flow at the PEF treated areas. **b** The increased flow almost returned to the baseline levels three weeks after PEF treatment. **c** Angiogenesis marker Nestin immunohistochemistry. Striking increase in the Nestin expression in the papillary dermis capillaries (black arrows) in comparison to untreated skin was observed from one day to three weeks after PEF. The intensity of staining two months after PEF was very similar to control, suggesting the maturation of the vessels. **a-c** Five animals were used for each time point. Error bars show ± SEM. **d** Secretion of pro-angiogenesis factors to the PEF treated area of the skin. All samples were normalized to the total protein. The bars show fold increase at the treated areas in comparison to expression levels in untreated animals. The bars show the average from measurements between 6 treated areas in 3 different animals. Error bars show ± SEM. (*) p-val < 0.05. Statistical analysis was performed by first using one-way ANOVA for multivariate analysis and post hoc using Dunnett’s tests to assess significance between individual groups. Significance was set at *P* < 0.05.

Application of 200 pulses at 500 V, 70 μs, 3 Hz seemed to eliminate the microcirculation at the treated area (Fig. 4a). The flow, however, was restored to baseline six hours after treatment (Fig. 4a,b). Twelve hours after PEF administration, the detected flow increased more than 42 ± 17% in comparison with baseline levels (Fig. 4a). These flow changes were local and were confined to the area of the skin where the electrodes were positioned during PEF administration. This increased flow in the center of the PEF-treated area reached a maximal level of 133 ± 20% three days after PEF administration (Fig. 4b). The flow was still 100 ± 25% higher in the treated area 1 week after PEF application, but reduced to baseline levels three weeks after treatment (Fig. 4b).

Immunohistochemical staining for Nestin, a marker for angiogenesis^26^, showed increased staining intensity in the papillary dermal microvasculature, detected from one day to three weeks after PEF treatment (Fig. 4c). In contrast to untreated skin, analysis of released cytokines and growth factors showed significantly increased levels of multiple factors known to control angiogenesis, including MCP-1^27^,VEGF^28^,IP-10^29^,IL-10^30^, EGF^31^, and Il-1b^32^ (Fig. 4d).

### Pulsed electric fields trigger increased skin metabolism

**Figure 5:**
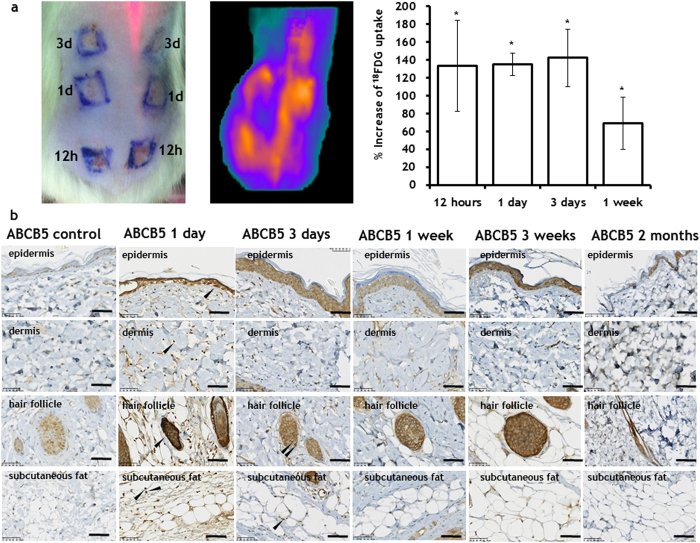
Increased skin metabolism after PEF administration. The increased metabolism of skin areas treated by PEF was detected using both **a** ^18^FDG uptake (Top panel) and **b** Increased expression of ABCB5, between 1 day to 3 weeks after PEF treatment. The increased expression of ABCB5 was observed in subpopulations of keratinocytes, cells in the dermis, cells in the hair follicles (in various parts of the follicle) and in subcutaneous fat. For ^18^FDG measurements, three rats were used with two treated areas per time point. Error bars show ± SEM. For ABCB5 expression, 5 rats were used for each time point. (*) p-val < 0.001. Statistical analysis was performed by first using one-way ANOVA for multivariate analysis and post hoc using Dunnett’s tests to assess significance between individual groups. Significance was set at *P* < 0.05.

Active skin rejuvenation mobilizes multiple reactions, and hence, the induction of rejuvenation should increase the total skin metabolism. To assess the impact of PEF on total skin metabolism, we measured glucose uptake by PEF-treated areas of the skin relative to normal untreated skin, and also performed immunohistochemistry for ATP transporter ABCB5. To quantify glucose uptake, we used positron emission tomography (PET) imaging with 2-Deoxy-2-[^18^F]fluoro-D-glucose (FDG). We found elevated glucose uptake in the PEF-treated areas of the skin for all studied time points, with maximum uptake observed during the first three days after PEF administration. In particular, 12, 24, and 72 hours after PEF, the treated areas exhibited increased FDG uptake at nearly the same level of 133 ± 50%, 135 ± 50%, and 142 ± 32%, respectively, as compared to the normal, untreated skin in the same animal (Fig. 5a). However, one week after treatment, FDG uptake in the PEF treated skin diminished to 69 ± 30% (Fig. 5a, Fig. S5).

Twenty-four hours after treatment, a significant overexpression of ABCB5 was observed in the PEF-exposed cells, including epidermis, dermis, hair follicles, and fat (Fig. 5b). This overexpression decreased during the first three weeks following treatment (Fig. 5b). Two months after PEF administration, the expression of ABCB5 in the epidermis, dermis, hair follicles and fat was very similar to that of untreated skin.

### Electro-thermal modeling of electric fields distribution and temperature changes in various skin layers during application of PEF

**Figure 6:**
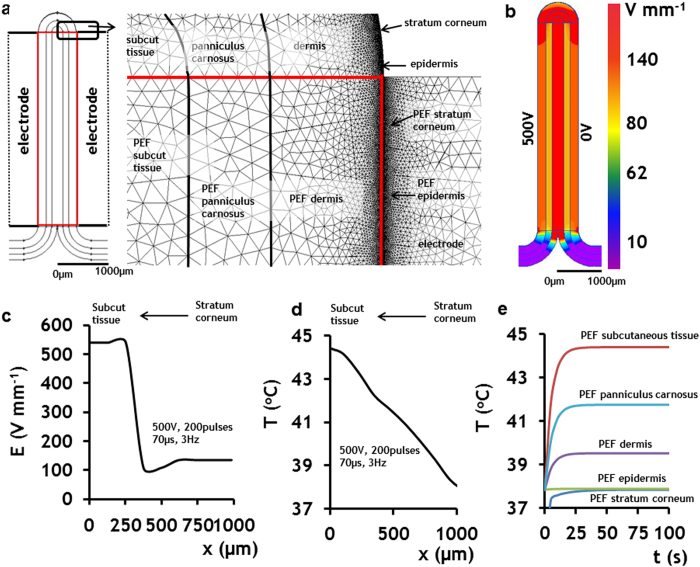
Electro-thermal model of PEF applied at the rat skin. The figure shows the electro-thermal model when the applied PEF protocol was 500 V, 70 μs pulse duration, and 200 pulses delivered at 3 Hz. **a** Finite elements numerical model, and geometry and solved mesh of the rat skin with all major composing layers. The skin is divided into two regions: 1) untreated skin and 2) PEF-treated skin. PEF-treated skin layers are tagged with PEF sign. **b** 2D map of electric field distribution in the skin slice. **c** The simulated values of electric field strengths in various skin layers (right panel). **d** Final simulated temperature distribution immediately after PEF. **e.** Time-dependent temperature increase in various skin layers during PEF administration.

Skin is an extremely heterogeneous organ, not only in terms of cell types, but also in terms of electrical conductivity. Our experimental results show that PEF have a variety of biochemical and microstructural effects on epidermal and dermal layers. Each layer of the skin has its own electrical conductivity, which affects the distribution of the electric fields and thus predicates the local electric field intensity according to Poisson’s equation (Eq.1). To study the electric field distribution within the various layers in rat skin, we constructed a numerical model that simulates distribution of electric fields in the skin using the Finite Element Methods (FEM). We modeled the geometry and electrical properties of normal rat skin positioned between two plate electrodes – the configuration used in our PEF experimental system (Fig. 1a). The geometry of the model, which includes the electrodes and skin composed of various layers, is shown in Fig. 6a.The electrical and thermal properties of the skin layers used for modeling appear in Table 3^33^,^34^. In this work we divided the modeling in two independent problems. In the model, we modeled the distribution of the electric fields in the skin as observed at the end of electroporation. In the second model, we assumed that PEF provide constant power to the skin and thus we solved a separate model for tissue heating as described below.

Healthy skin tissue has both electrical resistance and capacitance properties. However, the charging time of the capacitor component of healthy skin is very small in comparison to pulse duration^35^. Therefore, we used a direct current (DC) conductance electric model to calculate the distribution of the electric fields in heterogeneous skin. In our 2D model, we used the following Equation 1 to calculate the local electric field strength in each point of the skin:
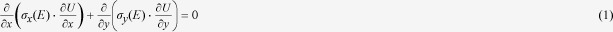
where *σ* is the electrical conductivity (S m^−1^), and *U* (V) is the electric potential, x and y are coordinates of a unit vector.
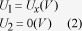


FEM allows study of the electric field distribution in the complex geometry of objects with different electric properties. We simulated the electric field distribution in different skin layers under the following boundary conditions applied on the electrodes:
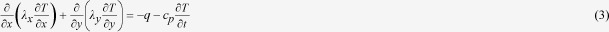
where *U*_*x*_is the potential applied on the cathode and *U*_2_ (0 V) is the potential on the grounded anode. *U*_*x*_ was set to 100, 200, 300, 400, and 500 V at different simulations.

The 2D map of electric field distribution in the cross-section of the rat skin exposed to 500 V, 200 pulses, with 70 μs duration delivered at 3 Hz is shown in Fig. 6b. At the steady state, the electric field exposure of the cells in the epidermis and dermis was 134 Vmm^−1^, muscle fibers in the panniculus carnosus - 107 Vmm^−1^, and cells in the subcutaneous tissue - 540 Vmm^−1^ (Fig. 6c).

The electric field distribution at various skin layers under additional boundary conditions of the applied voltage is shown in Fig. S1c. The five panels on the left show the 2D map of the electric field distribution. The plot on the right shows the numerical values of the electric field at different layers of the skin as a function of voltage applied on the cathode. The model shows that there is a drop in the electric field strength in the skin layers (stratum corneum, epidermis, and dermis) close to the electrodes. An additional drop occurs in the dermal muscle - panniculus carnosus. The largest magnitude of the electric field can be observed in the subcutaneous tissue. The electric field strength here is 4- and 5-fold higher than that in the epidermal and dermal layers and panniculus carnosus, respectively.

Using FEM we also modeled the time dependent temperature distribution in the skin treated by PEF. To calculate the temperature increase resulting from the PEF application, we solved the transient heat transfer problem using the following Equation 3:
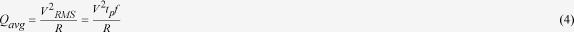
where *T* is temperature (K), *λ* (W K^−1^ m^−1^) is thermal conductivity, *c*_*p*_(J K^−1^ kg^−1^) is specific heat capacitance, *t*(s) is time, and *q(*W m^−3^) is volume power of heat sources. In our problem, *q is* the average volume power supplied to the tissue by pulsed electric fields. The following equation describes the calculation of power supplied by a square pulsed electric field:
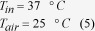
where *Q*_*avg*_*(W)* is the total average power delivered by a square pulsed electric field, *R* (ohm) is resistance, *V*_*RMS*_ is the root mean square voltage, *V* (V) is applied voltage, *t*_*p*_(s) is pulse length, and *f*(Hz) is the frequency of the pulse wave. The boundary conditions used to solve Equation 4 are:
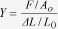
where *T*_*in*_ (°*C)* is the initial and internal temperature of the body and *T*_*air*_ (°*C)* is the constant temperature of the air. We assume that heat is transferred by convection between the surfaces of the body, the electrodes, and the air. We also assume that the convection coefficient between skin surface, electrode surface and the air is 5 (W K^−1^ m^−2^)^36^.

The simulation results for 200 pulses of 70 μs duration each, delivered at 500 V and 3 Hz are shown in Fig. 6d,e. The largest increase in temperature is observed in the subcutaneous tissue. Figure 6d shows the spatial distribution of the temperature in the treated skin at the end of PEF administration. Figure. 6e shows time dependence of temperature in different skin layers. Remarkably, all layers display temperature increase during the first 45 seconds followed by a steady-state condition. The maximum temperature at the steady-state level in the stratum corneum and epidermis, dermis, panniculus carnosus and subcutaneous tissue was found to be 37.8 °C, 39.5 °C, 41.5 °C, and 44.3 °C, respectively.

## Discussion

The demand for skin rejuvenation technologies will continue to grow with the increasing aging population. In this work we introduced a novel method for inducing skin rejuvenation using high voltage, short, non-thermal pulsed electric fields. PEF has been in the focus of skin therapies for molecules delivery^16^.To the best of our knowledge, this is the first report on rejuvenation potential of PEF for skin therapies. The procedure was tolerated well by animals. However translation of the method to humans will require further studies and device development, in which pain reduction is necessary^37^,^38^.

From all the advantages of PEF in contrast to other rejuvenation approaches, the most important one is that PEF intervention occurs on the cellular level, affecting only cell membranes while preserving the extracellular matrix architecture. The major PEF-induced changes in the skin contributing to rejuvenation are summarized in Table 4. One of the major challenges with the translation of PEF technologies to clinics is muscle contraction. Several approaches to address this challenge proposed to focus electric fields in the region of interest, avoiding non-target tissue and to use high frequency of pulse delivery^39^,^40^. We expect, however, that the magnitude of PEF required for skin rejuvenation will be lower than used in those studies for tissue ablation; therefore, further reducing the contractions.

Importantly, the mechanism, or combination of mechanisms, by which PEF affects cells is not completely understood. Partial necrosis due to cell leakage, apoptosis due to calcium influx, cell membrane reversible and irreversible electroporation, oxidative damage to the membrane, local pH changes, changes caused by reactive oxygen species, and other mechanisms have been proposed in the last four decades and are currently under scrutiny^16^.

Our results demonstrate that PEF administration induces skin re-epithelialization characterized by keratinocyte proliferation and growth at the sites where epidermis was focally ablated (Fig. 2b). PEF lead to focal irreversible electroporation of keratinocytes, while the mechanical properties of the skin, stratum corneum, and the extracellular matrix are not affected in the long-term^17^. This specific targeting of cells and preservation of the skin’s acellular structures is a unique property of PEF that differentiates it from other physical skin rejuvenation methods. We showed that PEF treated epidermis was temporarily thickened. This thickening correlated with the overexpression of p63 in the subpopulation of uninjured keratinocytes, suggesting that the activation of epidermal stem cells was involved in the regeneration/thickening process. Importantly, the thickening of the epidermis was temporal, and the thickness returned to baseline two months after the treatment. Consequently, PEF represents a noninvasive method to control keratinocyte activation and can provide a very convenient and chemical-free therapy for promoting re-epithelialization.

The observed prominent epidermal growth phenomenon can be partially explained by the molecular changes in the PEF-treated areas of the skin. PEF causes prominent changes in the levels of various cytokines and growth factors in the treated areas (Fig. S4). The released factors could bind to the preserved cell-free extracellular matrix. This, in turn, could lead to their increased local concentrations, and subsequently expose cells to higher levels of these factors. The increased levels of some of these factors such as IL-6^22^, EGF^22^, and VEGF^23^ were previously shown to induce keratinocyte proliferation and migration. Additional support of the observed prominent epidermis proliferation comes from the upregulation of skin metabolism as a result of PEF administration. This is demonstrated by the increased glucose uptake and overexpression of ATP transporter ABCB5 (Fig. 5). The latter is responsible for waste transport outside the cells, whereas increased glucose consumption has direct association with intense proliferation of epidermis, which occurs during the first 3 days after PEF treatment (Figs. 2c and 5). At the same time, ABCB5 is an established marker for skin cancer^41^. Hence, the absence of ABCB5 positive cells two months after PEF administration supports the tumor-free skin regeneration process (Fig. 5b). In the previous work^17^, we reported on the increased infiltration of neutrophils and leucocytes in the panniculus carnosus subregion of PEF treated skin 24 h after treatment. The role of these cells in skin rejuvenation is complex and currently is under investigation by our groups.

PEF induced skin collagenesis and led to the temporally increased fiber density in the skin, without the induction of scar formation (Fig. 3). Unchanged directional variance of the fibers two months after PEF administration (as confirmed by a dermatopathologist’s morphological analysis), indicates the absence of scar formation at the treated area^24^. IL-6^42^,EGF^43^,IP-10^43^, MCP-1^43^, and IL-10^43^, which were shown in literature to regulate collagen production, were significantly upregulated in the PEF-treated skin in comparison to the untreated skin (Fig. S4). In addition, our results also showed that the increase in fiber density, which was observed three days after PEF administration, was still observed three weeks after treatment. One of the limitations of this study is that the turnover rate of collagen may differ in human skin. However, previous studies showed that some properties of rat and human skin collagen maturation rate change with the same rate with age^44^. An additional limitation of this study is the focused use of animal models with healthy skin. Nevertheless, we anticipate that the knowledge acquired in this study to be essential for the development of PEF therapies for diseased skin; for it provides a methodology for PEF parameters optimization, enabling one to obtain a desired effect from PEF. Our future work will focus on the administration of PEF for old rat skin (to assess rejuvenation), diseased rat skin (to assess disease response), and wounded rat skin (to assess healing improvement) and will be based on the results of this study. However, the response of human damaged skin to PEF administration may similarly be different from what we find in animals, and additional optimization of PEF protocols may be required.

We also have investigated the impact of principal components of PEF therapy—namely, electric field strength (correlated with the applied voltage), number of pulses, and pulse duration—on dermal collagenesis induction. Both the relative contribution and dose response of each of the investigated parameters have been evaluated individually. For acute collagenesis, pulse length had the highest impact, applied voltage had average impact, and pulse number had the lowest impact (Table 1). For long-term collagenesis, the disposition of these parameters’ roles was somewhat opposite. Pulse number had the most prominent impact, while the applied voltage and pulse lengths had similar effects (Table 2). Dose response analysis for each individual parameter of PEF showed that the increase in voltage had the most significant effect on collagenesis both three weeks and two months after treatment (Fig. 1b,e). At the same time, our statistical analysis did not show significant impact of the increase of pulse duration and number of pulses on collagenesis (Fig. 1c,d,f,g). These observed differences can be explained via different mechanisms by which each of the PEF parameters affects the cells. In particular, electric field strength was shown to correlate with the total area of cell membrane becoming permeable to specific molecules, whereas the number of pulses further increased permeability of this area^45^. Though the precise molecular mechanisms relating cell membrane electroporation with collagen synthesis are currently unknown, our data suggests the possibility of such a relationship. An addition explanation to the not-stray forward results from the dose response study may be the variation in response time of various cells to the various components of PEF treatment.

Insufficient blood supply is a common problem in damaged skin, and multiple previous studies have demonstrated that blood supply improvement increases wound-healing rates, prevents infection, and restores skin function. In this respect, it is important to note that, according to our results (Fig. 4), PEF promotes blood flow through the treated area up to one week after the treatment. The initial vasoconstriction of the skin vessels (Fig. 4) after the application of electric fields have been reported previously in the literature. This vasoconstriction phenomena was thought to be mediated by the sympathetic nervous system at the low doses, or by increased interstitial pressure and decreased intravascular pressure due to irreversible damage to vessels that lead to leakage at the high doses of the PEF^46^,^47^,^48^. No long-term increase of the skin perfusion after PEF been reported before this study to the best of our knowledge. This is likely due to the formation of new vessels, as detected by Nestin immunohistochemistry. The presence of Nestin-expressing cells in the microvasculature suggests activation of dermal stem cells from the hair follicle lineage^26^. Enhanced glucose uptake and upregulation of ABCB5 expression indicate increased metabolism and, most likely, the appearance of progenitor cells^49^ at PEF treated sites. Additionally, elevated levels of MCP-1^27^,VEGF^28^,IP-10^29^,IL-10^30^, EGF^31^, and Il-1b^32^ (Figs. 4 and S4) further confirm the induction of angiogenesis at the PEF treated sites. Importantly, the observed increased levels of angiogenesis-regulating factors were temporary, and reduced to the basal levels three weeks after the PEF treatment. This transient nature of PEF-induced angiogenesis implies that it is a controllable process—a crucial fact for the future development of PEF-based therapies; for an opposite process—namely, uncontrollable induction of angiogenesis—can lead to tumor formation, which is considered a major drawback of various potential angiogenic drugs. Consequently, consistent with the notion that PEF-induced angiogenesis is controllable, it is significant to note that no tumor formation was observed in any of our studies.

Our numerical models, developed for studying specific effects of electric fields on cells located in different skin layers, provided an intriguing picture of electric field distribution in heterogeneous tissues. We showed that skin heterogeneity in structure and conductivity determines the local values of electric fields acting on the cells (Fig. 6 and S1). These results are of particular importance, as they allow for precisely targeted therapies of the skin and other organs. They also provide valuable information for the other pulsed electric field applications in medicine such as electrochemotherapy, electrogenetherapy, DNA vaccination, and irreversible electroporation; for the results indicate which population of skin is exposed to the specific strengths of the electric fields, a factor which controls the efficacy of all these therapies.

Our numerical modeling also implies that the temperature distribution in the skin exposed to PEF is not homogeneous, and rather depends on the thermal and electrical properties of the particular skin layer (Fig. 6d,e). Moreover, the heat conductance properties of a tissue depends upon the blood flow and the specific value of electric field^19^. In this paper we used the average properties of the tissue skin as a first approximation to the dynamic changes in the thermal and electric conductivity that take place during application of electric fields. Simulation results shown on Fig. 6d,e indicate that the largest increase in temperature is observed in the deep layers of the subcutaneous tissue. The simulated temperature increase lasts less than one minute. This temperature increase could possibly induce a weak heat shock response^50^, which in turn might further promote skin regeneration. The heating of tissue decreases from the inside out, so papillary dermis and epidermis are the least-heated areas of skin exposed to PEF (Fig. 6d). This transient heating modeling reveals interesting dynamics of skin heating during PEF, where the environmental boundaries of the treated skin region are included (Fig. 6e). During the application of PEF pulses, the skin temperature increases to only a specific maximal level (reached during the first 45 seconds for the studied PEF protocol), and then stabilizes at a new steady-state, equilibrated with the surrounding environment. These interesting results suggest that further increase in the number of pulses delivered at the applied frequency will not result in skin temperature elevation. Regardless of certain limitations of our model, such as approximate heat transfer coefficients and the effect of blood flow on tissue warming/cooling, these observations are anticipated to be of particular importance to multiple PEF-based therapies beyond the scope of this study, such as tumor ablation by non-thermal irreversible electroporation, and wound disinfection by PEF. Decoupling of tissue thermal properties changes due to the tissue permeabilisation and blood flow changes is a major challenge, solution of which would in the future provide a more detailed understanding of the processes involved.

To summarize, we developed a new non-invasive, non-thermal method of skin rejuvenation using high voltage, short Pulsed Electric Fields. PEF provide a promising low-cost, complication-free, and easy-to-adapt procedure resulting in the induction of prominent proliferation of the epidermis, formation of microvasculature, secretion of new collagen, and increased metabolic activity in treated skin as compared to untreated control skin. Our results suggest that PEF can significantly improve skin function and rejuvenation. In particular, de novo epidermis proliferation can improve skin barrier function against infection and hazardous materials. The induction of collagenesis can enhance skin mechanical properties and appearance. And, last but not least, the induction of angiogenesis can facilitate nutrient supply and waste removal from the skin, further improving skin health. Given these benefits and advantages over the existing approaches, we believe that PEF can potentially serve as a new non-invasive skin therapy for multiple patients.

## Materials and Methods

### Animals

6-week old female Sprague-Dawley rats (~200 g, N = 74) were purchased from Charles River Laboratories (Wilmington, MA). The animals were housed in individual cages with access to food and water ad libitum, and were maintained on a 12-hour light/dark cycle in a temperature controlled room. All animal procedures were approved by the Subcommittee on Research Animal Care (IACUC) of the Massachusetts General Hospital (protocol number 2012N000077) and were in accordance with the guidelines of the National Institutes of Health (NIH).

### Numerical Model

Numerical solutions for electric field distribution in skin and the thermal effects of electric fields were performed in QuickField (Terra Analysis, Denmark). The software files with the model appear as supplementary information. A viewer version of QuickField is available for free on http://quickfield.com/free_soft.htm (Accessed July 2014).

### Pulsed electric fields treatment

Prior to PEF administration, animals were anesthetized with isoflurane. Their fur was clipped along the dorsal surfaces and wet with the tap water. Subsequently, a designated area was subjected to PEF using contact electrodes with a surface area of 1 cm^2^. For the Taguchi Orthogonal array studies, the PEF parameters appear in Table 2S. Square pulses were delivered using BTX 830 pulse generator (Harvard Apparatus Inc., Holliston MA, USA). For the detailed long-term studies the following PEF protocol was applied: 500 V, 200 pulses, 70 μs pulse duration, 3 Hz. Currents were measured *in vivo* using PicoScope 4224 Oscilloscope with Pico Current Clamp (60 A AC/DC) and analyzed with Pico Scope 6 software (Pico technologies Inc.,UK).

### Laser Doppler Scanning

A laser Doppler imager (Moor Instruments, Wilmington, DE) was used to assess blood flow. The laser Doppler source was mounted on a movable rack exactly 20 cm above the dorsum of the rat after the animal was anesthetized and restrained on the underlying table. The laser beam (780 nm) reflected from circulating red blood cells in capillaries, arterioles, and venules was detected and processed to provide a computerized, color-coded image. By using image analysis software (Laser Doppler Perfusion Measure, Version 3.08; Moor Instruments), mean flux values representing blood flow were calculated from the relative flux units for the areas corresponding to the dorsum of the rats. The analyzed region of interest was 0.25 × 0.25 cm. Baseline images were obtained from each rat before treatment was administered. Then, the rats were treated by PEF, and serial laser Doppler images were obtained subsequently. We compared the mean flux values in the center of the PEF treated area with the basal level at the same spot.

### Skin mechanical properties measurement

To assess skin mechanical properties, we performed tensile mechanical testing on excised samples. Skin at the PEF treated sites (1 cm^2^) was excised at 1 day, 3 days, 1 week, 3 weeks and 2 months (N = 5 per time point). Skin was placed in the standard saline solution before tension measurement. Tension measurements were performed within 1 hour after the excision. Before measurement, thickness of each sample was measured. Skin samples were mounted on the eExpert4000 tensiometer (ADMET, MA). The load was applied using 1000 g-Tension-1344004 transducer. Stress at extension type of analysis was chosen on the MTESTQuattro software (ADMET, MA). Stress was applied using a ramp waveform to cause a 2 mm/min extension rate. The elongation/stress data was exported to Microsoft Excel ver.7. After the acquisition of force-elongation measurements, we constructed the stress-strain curve of the rat’s skin. Young’s modulus was extracted from the linear part of the stress-strain curve using the following equation:
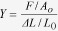
where *Y* (Nmm^−2^) is the elastic Young’s modulus, *F (N*) is the applied force, *A*_*0*_ is the cross-section of the skin samples, *ΔL* (mm) is the elongation of the skin sample under force, and *L*_*0*_(mm) is the original length of the tested sample.

### Histology

Specimens were harvested 1 day, 1 week, 3 weeks, and 2 months following the initial PEF administration. At least five animals were euthanized for each time point. Three animals were used as controls. Skin samples were fixed in 10% formalin, embedded in paraffin, and cut into 7-um sections. Sections were stained with hematoxylin and eosin (H&E), Herovichi, and Masson’s Trichrome. Tissues were processed and stained by the Rodent Histopathology Core at Harvard Medical School. Slides were evaluated by three separate investigators, including an experienced dermatopathologist in a blinded fashon. Color images of each entire tissue section were acquired using NanoZoomer Digital Pathology System (Nanozoomer 2.0-HT slide scanner (Hamamatsu, Hamamatsu City, Japan).

### Immunohistochemistry of rat skin tissue

7 μm paraffin-embedded tissue sections on glass slides were baked a 56 °C for 30 minutes, followed by deparaffinization in xylene and rehydration in graded alcohol into water. Antigen retrieval was performed by boiling the slides in 10 mM Sodium Citrate buffer pH = 6.0 for 30 minutes. Tissue sections were permeabilized with 0.1% Triton X-100. Endogenous peroxidase activity was quenched with 3% hydrogen peroxide in 60% methanol for 30 minutes. Biotin activity was blocked with Avidin-Biotin blocking reagent kit (Life Technologies, Grand Island, NY, 004303). Nonspecific proteins were blocked with 5% horse serum in PBS. Tissue sections were incubated with 1:200 dilution of the following mouse monoclonal antibodies that recognize the following proteins; p63 (Biocare, Concord, CA CM163 A), ABCB5 (Abcam, Cambridge MA, 140667), and Nestin (Abcam, Cambridge MA, 6142) in 5% horse serum overnight at 4 °C inside a humidified chamber. After washing, slides were incubated with horse anti-mouse biotinylated secondary IgG at 1:300 dilution (Vector laboratories, Burlingame, CA) for 60 minutes at room temperature, followed by incubation with ABC reagent (Vector Laboratories, Burlingame, CA PK6100) for 30 minutes. Specific protein signal was visualized by reacting the slides with 3,3′-Diaminobenzidine substrate in the presence of hydrogen peroxide in reaction buffer (Vector Laboratories, Burlingame, CA SK4100). Tissue sections were briefly counter-stained with Gill hematoxylin followed by rinsing in 0.1 M Ammonium Hydroxide (Fisher Scientific A669-500) for 30 seconds. Slides were briefly dehydrated and then mounted with Histomount solution (Life Technology, Grand Island, NY 008030). Color images of each entire tissue section were acquired using NanoZoomer Digital Pathology System (Nanozoomer 2.0-HT slide scanner (Hamamatsu, Hamamatsu City, Japan). The slides were stained in one batch to eliminate artifactual variations.

### Quantification of epidermis thickness and the relative levels of p63 expression

To quantify the effects of PEF on the thickness of epidermis, we measured the distance between basement membrane and stratum corneum in at least 25 points at 5 histologial sections obtained from different animals sacrificed at the same time point. To estimate the levels of p63 expression in the epidermis, we analyzed 5 fields of view per slide for each animal (5 animals per time point) at the highest possible resolution (80X). We analyzed the intensity of staining from 10 adjusted cells in each field of view. Distance measurement and intensity counts were conducted using ImageJ (NIH, MD, USA).

### Cytokines/chemokine/growth factor determination

Tissue was harvested at 2 and 5 weeks after PEF treatment, immediately flash frozen in liquid nitrogen and kept at −80 °C. The center of the treated area was excised and proteins were extracted in CelLytic^™^ MT Cell Lysis Reagent, C3228 (Sigma, MO) mixed with Protease Inhibitor Cocktail P8340 (Sigma, MO) using Mini-Beadbeater-1 (Biospec, OK) with 5.5 g/cc density zirconia beads (Biospec, OK). Tubes with the buffer and beads were shakes 4 times for 15 seconds with 1-minute intervals, when the tubes were kept on ice. Immediately after the extraction, total protein was quantified using 660 nm Protein Assay (Pierce,IL). All samples were than diluted to a single concentration. Quantification of cytokines, chemokines and growth factors was performed using MILLIPLEX MAP Rat Cytokine/Chemokine Magnetic Bead Panel and TGF-beta 3-Plex Array (Eve Technologies, Calgary, AB, Canada). After quantification, the concentration of each factor was normalized to the total protein concentration from the same sample.

### Automated image analysis of Trichrome stain for fiber density and orientation

Fiber density and orientation were calculated from images of Masson’s Trichrome stained sections using previously established image-processing algorithms^24^. Briefly, collagen fibers were identified from the images where the ratio of blue-to-red intensities exceeded a value of 2. Local fiber density was determined by the relative amount of collagen-positive pixels within a 50 pixel radius. Fiber orientation surrounding each image pixel location was also computed, and directional statistics were employed to compute the local directional variance of the fibers within a 50 pixel radius. Directional variance provided a metric that was inversely proportional to the strength of fiber alignment in the average fiber direction. Subregions of 300 × 700 μm corresponding to the center of the PEF-treated tissue region were defined through blinded evaluation of the original Trichrome images, and the average fiber density and directional variance were computed from each subregion. For controls, we used the values from subregions far away from the PEF treated zone.

### Automated image analysis of Herovichi stain for uncrosslinked/crosslinked collagen

Similar to Trichrome images, adjacent Herovichi stained sections were imaged and analyzed using a custom-written Matlab code. First, adjacent Trichrome and Herovichi section images were registered with each other using a Fourier-based cross-correlation method, which enabled the Trichrome-derived collagen fiber mask to be applied to the colorimetric analysis of fibers in the Herovichi sections. Within the collagen-fiber positive pixel locations, the ratio of blue-to-red intensity was computed from the Herovichi images. Because new, uncrosslinked collagen III fibers were stained blue, while mature, crosslinked collagen I fibers were stained red in the sections, this ratio was taken as a measure of the relative age of collagen fibers, with a higher ratio indicating newer, uncrosslinked fibers. Similar to Trichrome-associated outcomes, the average ratio within a 300 × 700 μm subregion was computed for statistical analysis.

### PET/CT imaging of skin metabolism

2-Deoxy-2-[^18^F]fluoro-D-glucose (FDG) was used to study glucose uptake in the PEF-treated skin regions in Sprague-Dawley rats by PET. FDG is a glucose analog that is trapped intracellularly after being phosphorylated. Therefore, it has been extensively used as a marker of the cell metabolic activity. PEF treatments on the dorsal skin of three rats were performed at 12, 24, 72, and 168 hours before imaging. For the imaging study, each rat was anesthetized with sodium pentobarbital (35 mg/kg, IP injection). A heparinized 3” catheter (BD 387334) was inserted into the tail vein and connected with a T connector (Abbott 1157218). Then, the animal was positioned in the animal bed of the microPET scanner (see below). PET image acquisition started immediately with intravenous administration of FDG through the T connector cap (flushed with 0.5 mL of saline). Radionuclide dose: up to 2 mCi per animal. Volume: up to 1 mL. After or in between the PET image acquisition, a single CT scan was obtained. It was used for further anatomical referencing, PET and CT images co-registration, and PET data correction for scattering and attenuation of the emission photons in the body tissues. Supporting doses (10 mg/kg, SC) of sodium pentobarbital were given during the course of the imaging experiments as necessary to maintain full anesthesia.

Inflammation and angiogenesis in the PEF-treated skin area may result in the elevated blood perfusion and thereby increase initial FDG concentration in the ablated region. However, the FDG fraction retained in the cells is dictated by the current need for glucose entering a glycolysis metabolic pathway. To characterize biokinetics of FDG in the skin and differentiate the FDG cellular uptake, we performed the dynamic PET data acquisition for the entire imaging duration of 1 hour (Fig. S6). The dynamic PET image consisted of a set of 1 or 4 minute frames. The images were reconstructed using OSEM3D/MAP protocol and processed to obtain numerical data of the radioactivity uptake in the PEF-treated and untreated skin regions. To do that, a 3-D object (ellipsoid or parallelepiped) was hand-drawn inside the region of interest (ROI) in the PET image. The mean radioactivity concentration (expressed in nCi/cc) in the ROI was measured as a function of time after injection. This time dependence of the FDG concentration (Fig. S6) was consistent with a three-compartment kinetic model, with each compartment representing capillary blood, interstitial and cellular space with unphosphorylated FDG, and cell with trapped phosphorylated FDG. Initial (0-8 minutes) FDG uptake reflects extravasation and further equilibration of the unphosphorylated FDG in the interstitial and cellular compartment. The following clearance (10-40 minutes) reflects re-absorption of this FDG into the blood as a result of its systemic clearance. The ultimate steady-state (50-60 minutes) radioactivity concentration in the skin represents the level of FDG undergoing glucose metabolic pathways, thus becoming trapped in the cells. The average value of this level was calculated in normal and PEF-treated skin regions and tabulated. FDG uptake in the PEF-treated skin regions was then derived as a percentage fraction with respect to the uptake in the normal skin. Average data and standard deviations were further obtained across three animals.

Imaging was carried out using a custom PET/CT imaging system consisting of MicroPET Focus 220 PET scanner (Siemens Medical Solutions, TN) and CereTom NL 3000 CT scanner (Neurologica, MA, USA). The imagers are aligned and equipped with a custom imaging bed extending through both imagers along the alignment axis, ensuring reliable PET/CT image registration. Focus 220 works in 3D mode and features a 22 cm animal opening, with an axial field of view (FOV) at 7.6 cm and transaxial FOV at 19 cm. The scanner’s detection system provides a 1.5 mm spatial resolution for ^18^F. CereTom NL 3000 is a 6-slice tomograph with high-contrast resolution of 0.4 mm (developed for human head imaging in ICU). The image acquisition settings were: tube voltage 100 kV, tube current 5 mA, resolution 6 s/projection, axial mode with slice thickness of 1.25 mm. Image pixel size was set to 0.49 × 0.49 × 1.25 mm. The image sharpness was optimized to soft tissue.

Raw data acquisition and histogramming were carried out on a Dell Precision PWS690 Workstation (Dell, Inc., Round Lake, TX; 3 GB RAM and 4 Xeon 3.20 GHz processors running under a 32-bit Windows XP [Microsoft Corp., Redmond, WA]) using Siemens MicroPET Firmware/Software, release 2.5 (Siemens Medical Solutions, Inc., Malvern, PA). Image reconstruction was carried out on a raid server (17.9 GB RAM and 8 Xeon 2.4 Ghz processors running under Microsoft Windows Server 2003, Enterprise x64 Edition) using Siemens MicroPET Firmware/Software, release 2.4.5. All subsequent image processing and analysis was performed on the Inveon Research Workplace 3.0 workstation (Siemens Medical Solutions, Inc., Malvern, PA) running under 64-bit Windows XP. During raw data histogramming and image reconstruction, the corrections for isotope decay, detectors dead-time, random coincidences, and tissue attenuation were applied.

### Statistical Analysis

Statistical analysis was performed with Statistics toolbox in MATLAB, R2009b (MathWorks, Natick, MA, USA) and JMP Pro 10 (SAS Institute, Cary, NC). The reported data are means ± SEM. Statistical analysis was performed by first using one-way ANOVA for multivariate analysis and post hoc using Dunnett’s tests to assess significance between individual groups. Significance was set at *P* < 0.05.

## Author Contributions

A.G., W.J.A. and M.Y. designed the study. A.G., S.K., and G.F.B. conducted the *in vivo* experiments, V.B. and M.P. performed PET-CT imaging and image analysis, K.P.Q. and I.G. performed histological image processing, A.G., G.F.B., H.A. and M.W. performed Doppler Imaging, H.A. and M.W. performed immunohistochemistry, M.C.M. performed histopathological analysis, A.G. developed the FEM model and performed integrated data analyses, AG drafted the manuscript, S.K., V.B., K.P.Q., H.A., G.F.B., M.W., I.G., M.P., M.C.M., W.G.A. and M.Y. reviewed and approved the manuscript.

## Additional Information

**How to cite this article**: Golberg, A. *et al*. Skin Rejuvenation with Non-Invasive Pulsed Electric Fields. *Sci. Rep.* **5**, 10187; doi: 10.1038/srep10187 (2015).

## Supplementary Material

Supplementary Information

## Figures and Tables

**Table 1:** Ranking of the pulsed electric field parameters’ effects on collagen increase three weeks after pulsed electric field application, according to Taguchi analysis.

**Level**	**Voltage <S/N> 3weeks**	**Pulse Length (us) <S/N> 3 weeks**	**Number of pulses <S/N> 3 weeks**
1	11	15	18
2	14	11	14
3	15	18	13
4	19	12	16
5	18	20	15
Δ	8	9	5
**Rank**	**2**	**1**	**3**

**Table 2:** Ranking of the pulsed electric field parameters’ effects on collagen increase two months after pulsed electric field administration, according to Taguchi analysis.

**Level**	**Voltage <S/N> 3weeks**	**Pulse Length (us) <S/N> 3 weeks**	**Number of pulses <S/N> 3 weeks**
1	14	20	18
2	17	25	11
3	19	16	17
4	19	17	19
5	24	15	24
Δ	10	10	13
**Rank**	**2**	**2**	**1**

**Table 3:** Material properties used for Finite Element modeling of electric field distribution in the skin.

	**Geometry**	**Electrical conductivity σ (S m**^**−1**^)	**Thermal conductivity λ (W K**^**−1**^ **m**^**−1**^)	**Heat capacitance c**_**p**_**(J kg**^**−1**^ **K**^**−1**^)	**Density ρ(kg m**^**3**^)
**Normal skin**					
** Stratum corneum**	Thickness 5 μm	0.0000125	0.23	3300	1100
** Epidermis**	Thickness 5 μm	0.2	0.23	3300	1100
** Dermis**	Thickness 400 μm	0.2	0.45	3300	1100
** Panniculus carnosus**	Thickness 300 μm	0.4	0.5	3300	1100
** Subcutaneous tissue**	Thickness 300 μm	0.02	0.19	3300	1100

**Electroporated skin**					
** Stratum corneum**	Thickness 5 μm	1	0.23	3300	1100
** Epidermis**	Thickness 5 μm	0.8	0.23	3300	1100
** Dermis**	Thickness 400 μm	0.8	0.45	3300	1100
** Panniculus carnosus**	Thickness 300 μm	1	0.5	3300	1100
** Subcutaneous tissue**	Thickness 300 μm	0.2	0.19	3300	1100
** Electrodes**	1 cm^2^ surface area, 2 mm apart	1.45 × 10^6^	16	466	8000

**Table 4:** The effects of pulsed electric fields on major skin properties.

**PEF effects on skin**	**3 days**	**1 week**	**3 weeks**	**2 months**
Epidermis thickness	3.7-fold increase	4.5-fold increase	2.2-fold increase	normal
Collagen fibers density	41% increase	55% increase	43% increase	normal
Microcirculation	133% increase	104% increase	normal	normal
Tumorigenesis	absent	absent	absent	absent
Scarring	absent	absent	absent	absent

## References

[b1] Fernandes J. R. *et al.* Micro-mechanical fractional skin rejuvenation. Plast. Reconstr. Surg. 131, 216–23 (2013). 23357983 10.1097/PRS.0b013e3182789afa

[b2] Mengeaud V., Dautezac-Vieu C., Josse G., Vellas B. & Schmitt A.-M. Prevalence of dermatoporosis in elderly French hospital in-patients: a cross-sectional study. Br. J. Dermatol. 166, 442–3 (2012). 21787367 10.1111/j.1365-2133.2011.10534.x

[b3] No Title. Am. Soc. Aesthetic Plast. Surgery. 2014 Cosmet. Surg. Natl. Data Bank Stat . Available at http//www.surgery.org/sites/default/files/Stats2013_3.pdf. Accesses 14 April 2014. (2013).

[b4] Carney C. M. *et al.* A Comparative Study of Root Defect Coverage Using an Acellular Dermal Matrix With and Without a Recombinant Human Platelet-Derived Growth Factor. Journal of Periodontology 83, 893–901 (2012). 22149763 10.1902/jop.2011.110144

[b5] Cerqueira M. T. *et al.* Cell sheet technology-driven re-epithelialization and neovascularization of skin wounds. Acta. Biomater. 10, 3145–3155 (2014). 24650971 10.1016/j.actbio.2014.03.006

[b6] Huemer G. M. *et al.* Comparison of the effectiveness of gene therapy with transforming growth factor-beta or extracorporal shock wave therapy to reduce ischemic necrosis in an epigastric skin flap model in rats. Wound Repair Regen. 13, 262–8 (2005) 15953045 10.1111/j.1067-1927.2005.130308.x

[b7] Ennis W. J., Lee C. & Meneses P. A biochemical approach to wound healing through the use of modalities. Clin. Dermatol. 25, 63–72 (2007) 17276203 10.1016/j.clindermatol.2006.09.008

[b8] Ottomann C. *et al.* Prospective randomized trial of accelerated re-epithelization of skin graft donor sites using extracorporeal shock wave therapy. J. Am. Coll. Surg. 211, 361–367 (2010). 20800193 10.1016/j.jamcollsurg.2010.05.012

[b9] Weinheimer-Haus E. M., Judex S., Ennis W. J. & Koh T. J. Low-intensity vibration improves angiogenesis and wound healing in diabetic mice. PLoS One 9, (2014) 10.1371/journal.pone.0091355 PMC3950202 24618702

[b10] EL-Domyati M. & Medhat W. Minimally Invasive Facial Rejuvenation. Expert Rev. Dermatol. 8, 565–580 (2013).

[b11] Pettersson A. *et al.* Heterogeneity of the angiogenic response induced in different normal adult tissues by vascular permeability factor/vascular endothelial growth factor. Lab. Invest. 80, 99–115 (2000). 10653008 10.1038/labinvest.3780013

[b12] Avram M. M., Tope W. D., Yu T., Szachowicz E. & Nelson J. S. Hypertrophic scarring of the neck following ablative fractional carbon dioxide laser resurfacing. Lasers Surg. Med. 41, 185–188 (2009). 19291746 10.1002/lsm.20755 PMC2747732

[b13] Cox S. E. & Adigun C. G. Complications of injectable fillers and neurotoxins. Dermatol. Ther. 24, 524–36 (2011). 22515668 10.1111/j.1529-8019.2012.01455.x

[b14] Golberg A. & Yarmush M. L. Nonthermal irreversible electroporation: Fundamentals, applications, and challenges. IEEE Transactions on Biomedical Engineering 60, 707–714 (2013). 23314769 10.1109/TBME.2013.2238672

[b15] Prausnitz M. R., Edelman E. R., Gimm J. A., Langer R. & Weaver J. C. Transdermal delivery of heparin by skin electroporation. Biotechnology (N. Y) . 13, 1205–9 (1995). 9636293 10.1038/nbt1195-1205

[b16] Yarmush M. L., Golberg A., Serša G., Kotnik T. & Miklavčič D. Electroporation-Based Technologies for Medicine: Principles, Applications, and Challenges. Annu. Rev. Biomed. Eng. 16, 295–320 (2014). 24905876 10.1146/annurev-bioeng-071813-104622

[b17] Golberg A. *et al.* Non-thermal, pulsed electric field cell ablation: A novel tool for regenerative medicine and scarless skin regeneration. Technology 1, 1–8 (2013). 24999487 10.1142/S233954781320001X PMC4078877

[b18] Rao R. S., Kumar C. G., Prakasham R. S. & Hobbs P. J. The Taguchi methodology as a statistical tool for biotechnological applications: A critical appraisal. Biotechnology Journal 3, 510–523 (2008). 18320563 10.1002/biot.200700201

[b19] Ivorra A., Al-Sakere B., Rubinsky B. & Mir L. M. *In vivo* electrical conductivity measurements during and after tumor electroporation: conductivity changes reflect the treatment outcome. Phys. Med. Biol. 54, 5949–5963 (2009). 19759406 10.1088/0031-9155/54/19/019

[b20] Truong A. B., Kretz M., Ridky T. W., Kimmel R. & Khavari P. A. p63 regulates proliferation and differentiation of developmentally mature keratinocytes. Genes. Dev. 20, 3185–3197 (2006). 17114587 10.1101/gad.1463206 PMC1635152

[b21] Pellegrini G. *et al.* p63 identifies keratinocyte stem cells. Proc. Natl. Acad. Sci. USA 98, 3156–3161 (2001). 11248048 10.1073/pnas.061032098 PMC30623

[b22] Hernández-Quintero M., Kuri-Harcuch W., González Robles A. & Castro-Muñozledo F. Interleukin-6 promotes human epidermal keratinocyte proliferation and keratin cytoskeleton reorganization in culture. Cell Tissue Res. 325, 77–90 (2006). 16550359 10.1007/s00441-006-0173-9

[b23] Wilgus T. A. *et al.* Novel function for vascular endothelial growth factor receptor-1 on epidermal keratinocytes. Am. J. Pathol. 167, 1257–1266 (2005). 16251410 10.1016/S0002-9440(10)61213-8 PMC1603795

[b24] Quinn K. *et al.* An automated image processing method to quantify of collagen fiber organization within cutaneous scar tissue. Exp. Dermatol. 24, 78–80 (2015). 25256009 10.1111/exd.12553 PMC4289465

[b25] Turner N. J., Pezzone M. A., Brown B. N. & Badylak S. F. Quantitative multispectral imaging of Herovici’s polychrome for the assessment of collagen content and tissue remodelling. J. Tissue Eng. Regen. Med. 7, 139–148 (2013). 22072426 10.1002/term.508

[b26] Amoh Y. *et al.* Nascent blood vessels in the skin arise from nestin-expressing hair-follicle cells. Proc. Natl. Acad. Sci. USA 101, 13291–13295 (2004). 15331785 10.1073/pnas.0405250101 PMC516562

[b27] Niu J., Azfer A., Zhelyabovska O., Fatma S. & Kolattukudy P. E. Monocyte chemotactic protein (MCP)-1 promotes angiogenesis via a novel transcription factor, MCP-1-induced protein (MCPIP). J. Biol. Chem. 283, 14542–14551 (2008). 18364357 10.1074/jbc.M802139200 PMC2386911

[b28] Neufeld G. & Kessler O. Pro-angiogenic cytokines and their role in tumor angiogenesis. Cancer and Metastasis Reviews 25, 373–385 (2006). 17006765 10.1007/s10555-006-9011-5

[b29] Bodnar R. J., Yates C. C. & Wells A. IP-10 blocks vascular endothelial growth factor-induced endothelial cell motility and tube formation via inhibition of calpain. Circ. Res. 98, 617–625 (2006). 16484616 10.1161/01.RES.0000209968.66606.10 PMC3826264

[b30] Dace D. S., Khan A. A., Kelly J. & Apte R. S. Interleukin-10 promotes pathological angiogenesis by regulating macrophage response to hypoxia during development. PLoS One 3, (2008) 10.1371/journal.pone.0003381 PMC2557127 18852882

[b31] Van Cruijsen H., Giaccone G. & Hoekman K. Epidermal growth factor receptor and angiogenesis: Opportunities for combined anticancer strategies. International Journal of Cancer 117, 883–888 (2005). 10.1002/ijc.21479 16152621

[b32] Kim G.-Y. *et al.* Proinflammatory cytokine IL-1beta stimulates IL-8 synthesis in mast cells via a leukotriene B4 receptor 2-linked pathway, contributing to angiogenesis. J. Immunol. 184, 3946–3954 (2010). 20194723 10.4049/jimmunol.0901735

[b33] Pavšelj N. & Miklavčič D. A numerical model of permeabilized skin with local transport regions. IEEE Trans. Biomed. Eng. 55, 1927–1930 (2008). 18595814 10.1109/TBME.2008.919730

[b34] Hasgall P. A., Neufeld E., Gosselin M., Klingenböck A. & Kuster N. IT’IS Database for thermal and electromagnetic parameters of biological tissues, Version 2.5, August 1st, 2014. www.itis.ethz.ch/database. (2014). Date of access: 19/02/2015.

[b35] Weaver J. C. Electroporation of cells and tissues. IEEE Trans. Plasma Sci. 28, 24–33 (2000).

[b36] Kurazumi Y. *et al.* Radiative and convective heat transfer coefficients of the human body in natural convection. Build. Environ. 43, 2142–2153 (2008).

[b37] Wallace M. *et al.* Tolerability of two sequential electroporation treatments using MedPulser DNA delivery system (DDS) in healthy adults. Mol. Ther. 17, 922–928 (2009). 19277016 10.1038/mt.2009.27 PMC2835142

[b38] El-Kamary S. S. *et al.* Safety and Tolerability of the Easy Vax^TM^ Clinical Epidermal Electroporation System in Healthy Adults. Molecular Therapy 20, 214–220 (2012). 22068424 10.1038/mt.2011.235 PMC3255586

[b39] Golberg A. & Rubinsky B. Towards electroporation based treatment planning considering electric field induced muscle contractions. Technol. Cancer Res. Treat. 11, 189–201 (2012). 22335414 10.7785/tcrt.2012.500249

[b40] Arena C. B. *et al.* High-frequency irreversible electroporation (H-FIRE) for non-thermal ablation without muscle contraction. BioMedical Engineering OnLine 10, 102 (2011). 22104372 10.1186/1475-925X-10-102 PMC3258292

[b41] Chartrain M. *et al.* Melanoma chemotherapy leads to the selection of ABCB5-expressing cells. PLoS One 7, (2012) 10.1371/journal.pone.0036762 PMC3360047 22675422

[b42] McGaha T. L. *et al.* Molecular mechanisms of interleukin-4-induced up-regulation of type I collagen gene expression in murine fibroblasts. Arthritis Rheum. 48, 2275–2284 (2003). 12905482 10.1002/art.11089

[b43] Kim M.-S., Song H. J., Lee S. H. & Lee C. K. Comparative study of various growth factors and cytokines on type I collagen and hyaluronan production in human dermal fibroblasts. J. Cosmet. Dermatol. 13, 44–51 (2014). 24641605 10.1111/jocd.12073

[b44] Le Lous M., Allain J. C., Cohen-Solal L. & Maroteaux P. The rate of collagen maturation in rat and human skin. Connect. Tissue Res. 9, 253–262 (1982). 6215209 10.3109/03008208209160271

[b45] Teissie J., Golzio M. & Rols M. P. Mechanisms of cell membrane electropermeabilization: A minireview of our present (lack of?) knowledge. Biochimica et Biophysica Acta. - General Subjects 1724, 270–280 (2005). 10.1016/j.bbagen.2005.05.006 15951114

[b46] Mandel Y. *et al.* Vasoconstriction by electrical stimulation: new approach to control of non-compressible hemorrhage. Sci. Rep. 3, 2111 (2013). 23828130 10.1038/srep02111 PMC3701318

[b47] Palanker D., Vankov A., Freyvert Y. & Huie P. Pulsed electrical stimulation for control of vasculature: temporary vasoconstriction and permanent thrombosis. Bioelectromagnetics 29, 100–107 (2008). 17918191 10.1002/bem.20368

[b48] Gehl J., Skovsgaard T. & Mir L. M. Vascular reactions to *in vivo* electroporation: characterization and consequences for drug and gene delivery. Biochim. Biophys. Acta. - Gen. Subj. 1569, 51–58 (2002). 10.1016/s0304-4165(01)00233-1 11853957

[b49] Frank N. Y. *et al.* Regulation of progenitor cell fusion by ABCB5 P-glycoprotein, a novel human ATP-binding cassette transporter. J. Biol. Chem. 278, 47156–47165 (2003). 12960149 10.1074/jbc.M308700200

[b50] Moloney T. C., Hoban D. B., Barry F. P., Howard L. & Dowd E. Kinetics of thermally induced heat shock protein 27 and 70 expression by bone marrow-derived mesenchymal stem cells. Protein Sci. 21, 904–909 (2012). 22505291 10.1002/pro.2077 PMC3403425

