# Supplementary material for: Skin Rejuvenation with Non-Invasive Pulsed Electric Fields
Source: Sci Rep. 2015 May 12;5:10187. doi: 10.1038/srep10187 (PMC4428072; doi:10.1038/srep10187)
Supplement: Supplementary Information [file srep10187-s1.pdf]

Supplementary information

Skin Rejuvenation with Non-Invasive Pulsed Electric Fields.

Alexander Golberg[1,2], Saiqa Khan[3], Vasily Belov[4], Kyle P. Quinn[5], Hassan Albadawi[6], G. Felix Broelsch[3], Michael T. Watkins[6], Irene Georgakoudi[5], Mikhail Papisov[4], Martin C. Mihm, Jr.[7], William G. Austen, Jr. [3], Martin L. Yarmush[1,8*]

1 Center for Engineering in Medicine, Department of Surgery, Massachusetts General Hospital, Harvard Medical School, and Shriners Burns Hospital, Boston, MA, 02114.
2 Porter School of Environmental Studies, Tel Aviv University, Tel Aviv, Israel.
3 Division of Plastic and Reconstruction Surgery, Massachusetts General Hospital, Harvard Medical School, Boston, MA 02114.
4 Department of Radiology, Massachusetts General Hospital, Harvard Medical School, and the Shriners Burns Hospital, Boston, MA, 02114.
5 Department of Biomedical Engineering, Tufts University, Medford, MA, 02155.
6 Division of Vascular and Endovascular Surgery, Massachusetts General Hospital, Harvard Medical School, Boston, MA 02114.
7 Department of Dermatology, Brigham and Women's Hospital, Harvard Medical School, Boston, MA 02115.
8 Department of Biomedical Engineering, Rutgers University, Piscataway, NJ 08854.

Taguchi analysis of individual impact of pulse amplitude, duration, and number on skin collagenesis.

The goal in these series of experiments was to determine the effects of pulsed electric field parameters on skin collagenesis. Pulsed electric field parameters include electric field amplitude (controlled by the applied voltage), pulse duration, number of pulses, and pulse frequency. The possible range of PEF parameters and their combinations is large. Therefore, to decrease the number of experiments but still be able to evaluate the impact of each parameter independently, we applied the Taguchi orthogonal array for the experimental design. We tested the impact of applied voltage (in the 100-500V range), pulse number (*N*) (200-1000 range) and pulse duration (t_p) (10-90µs range). We tested 5 levels of each of the parameters (**Table 1S**) towards increasing the collagen synthesis in the skin. **Table 2S** summarizes the experiments conducted for the L25 orthogonal Taguchi array, and is needed to determine the individual effects of each of the tested parameters on skin collagenesis. Three animals were used per each experimental design, with two repeats within the same animal (two sites on the animal skin were treated with the same PEF protocol).

To determine the effect each of the tested parameters had on the output, the signal-to-noise ratio (S/N) was calculated for each experiment. The calculation of S/N for each experiment was performed according to the Equations S1-S5, as follows:

$$S/N = 10\log\left(\frac{1}{N}\cdot\left(\frac{Sm1 - Ve1}{Ve1}\right)\right) \qquad \text{(S1)}$$

$$Sm1 = \frac{(\sum c_i)^2}{N} \qquad \text{(S2)}$$

$$Ve1 = \frac{Se1}{N-1} \qquad \text{(S3)}$$

$$Se1 = ST1 - Sm1 \qquad \text{(S4)}$$

$$Se1 = \sum c_i^2 \qquad \text{(S5)}$$

where c_i is the collagen concentration (mg/100mg tissue) measured in each experiments with *i* repeats and N is the total number of repeats for each experiment. The calculated S/N ratio for each experiment appears in Table 2S. Next, we calculated the average S/N value for each factor individually:

$$< S/N_{f,L} >= \frac{\sum S/N_{f,L}}{N_L} \qquad \text{(S6)}$$

Where *f* is the tested parameter (either voltage, or pulse duration, or pulse number) and *L* is the level as appears in **Table 1S**. To determine the effect of each parameter, we calculated the range:

$$\Delta = Max(< S/N_f >) - Min(< S/N_f >) \qquad \text{(S7)}$$

Highest ranking (1) was assigned to the parameters with the largest range. **Tables 1 and 2** summarize the individual responses for each of the tested parameters both three weeks and two months after the treatment, respectively.

Figure S1. Numeric simulation of electric field distribution in the rat skin under different boundary conditions. a. Schematic representation of the experimental setup and digital image of electrodes applied on the dorsal rat skin. **b.** Finite elements numerical model, geometry, and solved mesh of the rat skin with all major layers. The skin is divided into two regions: 1) normal skin and 2) PEF treated skin. PEF skin layers are tagged with PEF sign. **c.** Electric field distribution in different skin layers under various boundary conditions. The panels on the left show the 2D map of electric field distribution under various voltages applied on the electrode. The plot of the right shows the numerical values of the electric field strength at different skin layers under various boundary conditions.

Figure S2. PEF induced collagenesis. The graphs show experimental data on the total collagen detected in the skin treated by PEF with parameters described in Table 2S. Three animals were used for experimental groups, four animals were used for control, untreated skin. Error bars show ± SEM.

Figure S3. Hematoxylin and eosin stain (H&E stain) of rat skin 1 day after PEF administration. Arrows show areas of acellular epidermis and subcorneal and intraepidermial abscesses.

Figure S4. Secretion of cytokines, chemokines and growth factors after PEF administration. The changes in the expression of various signaling molecules were detected by MILLIPLEX MAP Rat Cytokine/Chemokine Magnetic Bead Panel and TGF-beta 3-Plex Array (Eve Technologies, Calgary, AB, Canada). Only detectible factors are shown. Each value is a result of averaging across three different rats. The results from quantification were normalized to the total protein. For values marked in red in the table and (*) in the plot, p-val<0.05. Error bars show ± SEM.

Figure S5. Digital photography and PET image of skin treated by PEF, one week after the treatment. The quantified results appear in Fig.6.

Figure S6. Time dependence of the FDG concentration in the PEF wounds. At least three animals were used. Error bars show ± SEM.

Table 1S. Tested levels for pulsed electric field parameters.

Level	Voltage	Pulse Length (us)	Number of pulses
1	100	10	200
2	200	30	400
3	300	50	600
4	400	70	800
5	500	90	1000

Table 2S. Experimental Array and Taguchi S/N ration for determination of critical pulsed electric field parameters: applied voltage, pulse duration, number of pulses.

Voltage (V)	Duration (μs)	Number of Pulses N	S/N (3weeks)	S/N (2months)
100	10	200	11	15
200	10	400	11	9
300	10	600	13	28
400	10	800	18	16
500	10	1000	23	34
100	30	400	10	12
200	30	600	12	16
300	30	800	7	25
400	30	1000	14	34
500	30	200	13	37
100	50	600	9	12
200	50	800	19	22
300	50	1000	14	13
400	50	200	28	16
500	50	400	18	18
100	70	800	10	18
200	70	1000	11	25
300	70	200	21	13
400	70	400	10	15
500	70	600	9	14
100	90	1000	14	13
200	90	200	18	12
300	90	400	19	17
400	90	600	23	16
500	90	800	27	16

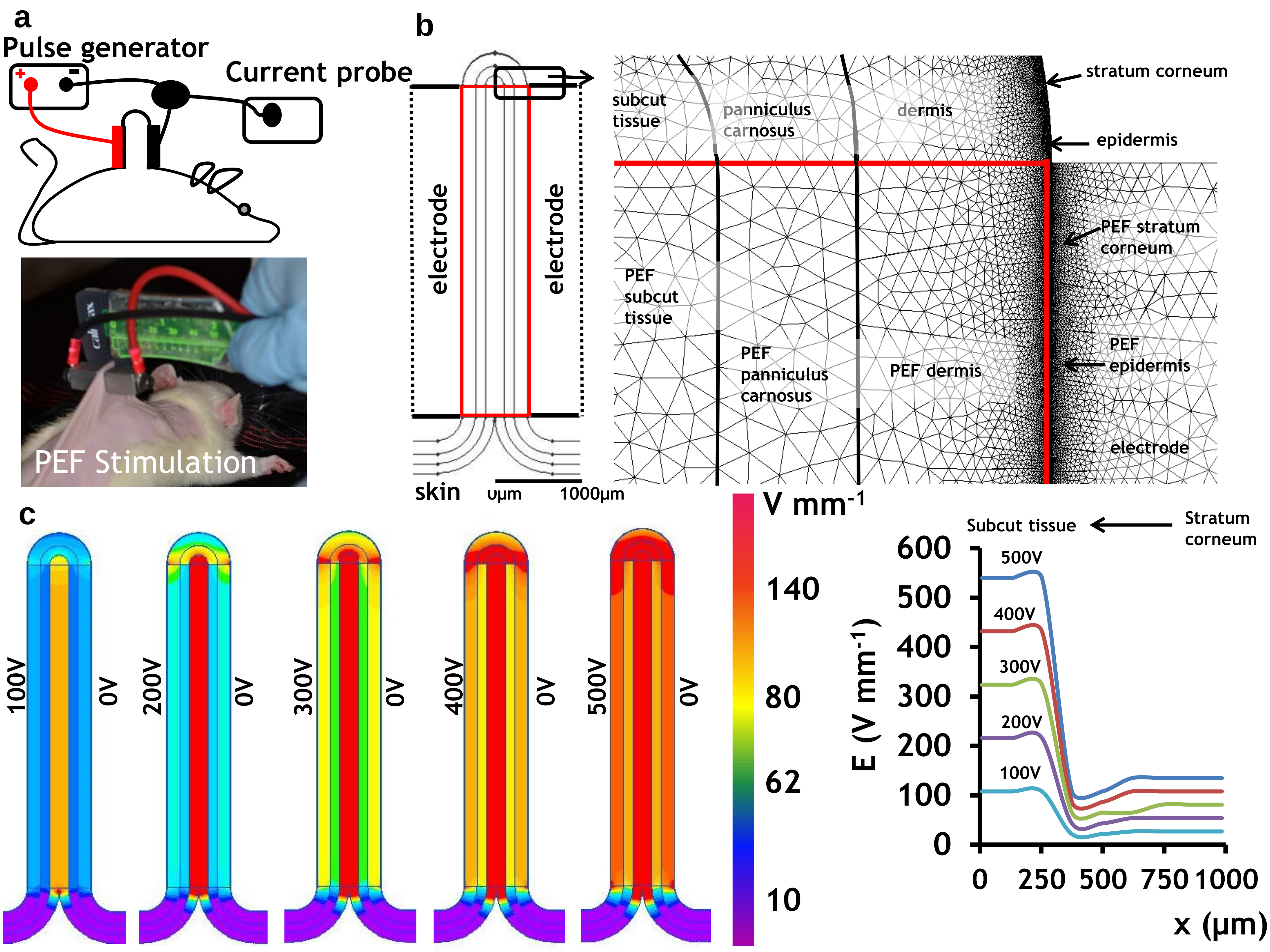

a
Pulse generator
Current probe
PEF Stimulation
b
electrode
electrode
skin
0μm
1000μm
subcut tissue
panniculus carnosus
dermis
stratum corneum
epidermis
PEF subcut tissue
PEF panniculus carnosus
PEF dermis
PEF stratum corneum
PEF epidermis
electrode
c
100V
0V
200V
0V
300V
0V
400V
0V
500V
0V
V mm-1
140
80
62
10
Subcut tissue
Stratum corneum
E (V mm-1)
600
500
400
300
200
100
0
500V
400V
300V
200V
100V
0
250
500
750
1000
x (μm)

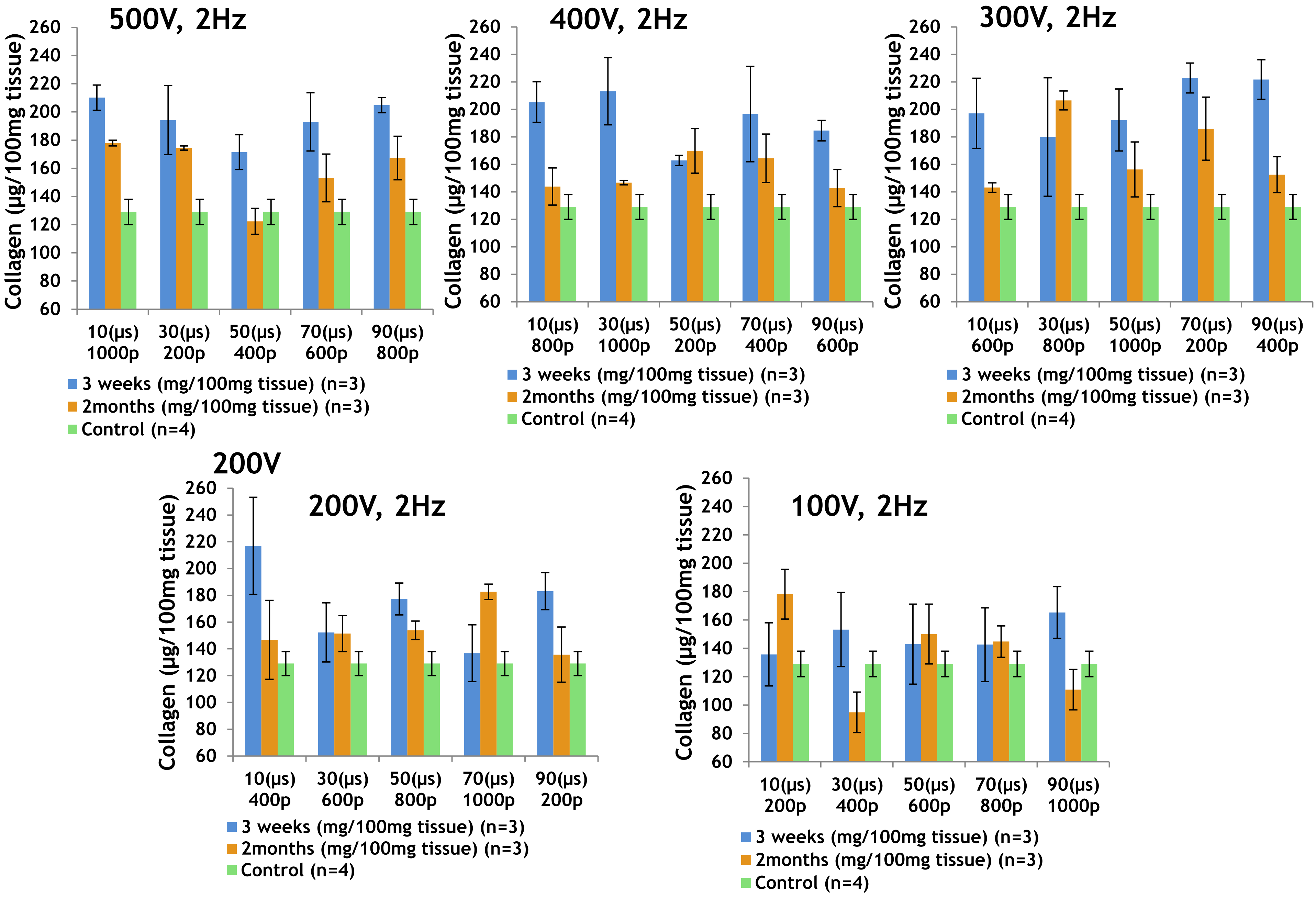
500V, 2Hz
400V, 2Hz
300V, 2Hz
200V
200V, 2Hz
100V, 2Hz
Collagen (µg/100mg tissue)
260
240
220
200
180
160
140
120
100
80
60
10(µs) 1000p
30(µs) 200p
50(µs) 400p
70(µs) 600p
90(µs) 800p
10(µs) 800p
30(µs) 1000p
50(µs) 200p
70(µs) 400p
90(µs) 600p
10(µs) 600p
30(µs) 800p
50(µs) 1000p
70(µs) 200p
90(µs) 400p
10(µs) 400p
30(µs) 600p
50(µs) 800p
70(µs) 1000p
90(µs) 200p
10(µs) 200p
30(µs) 400p
50(µs) 600p
70(µs) 800p
90(µs) 1000p
3 weeks (mg/100mg tissue) (n=3)
2months (mg/100mg tissue) (n=3)
Control (n=4)

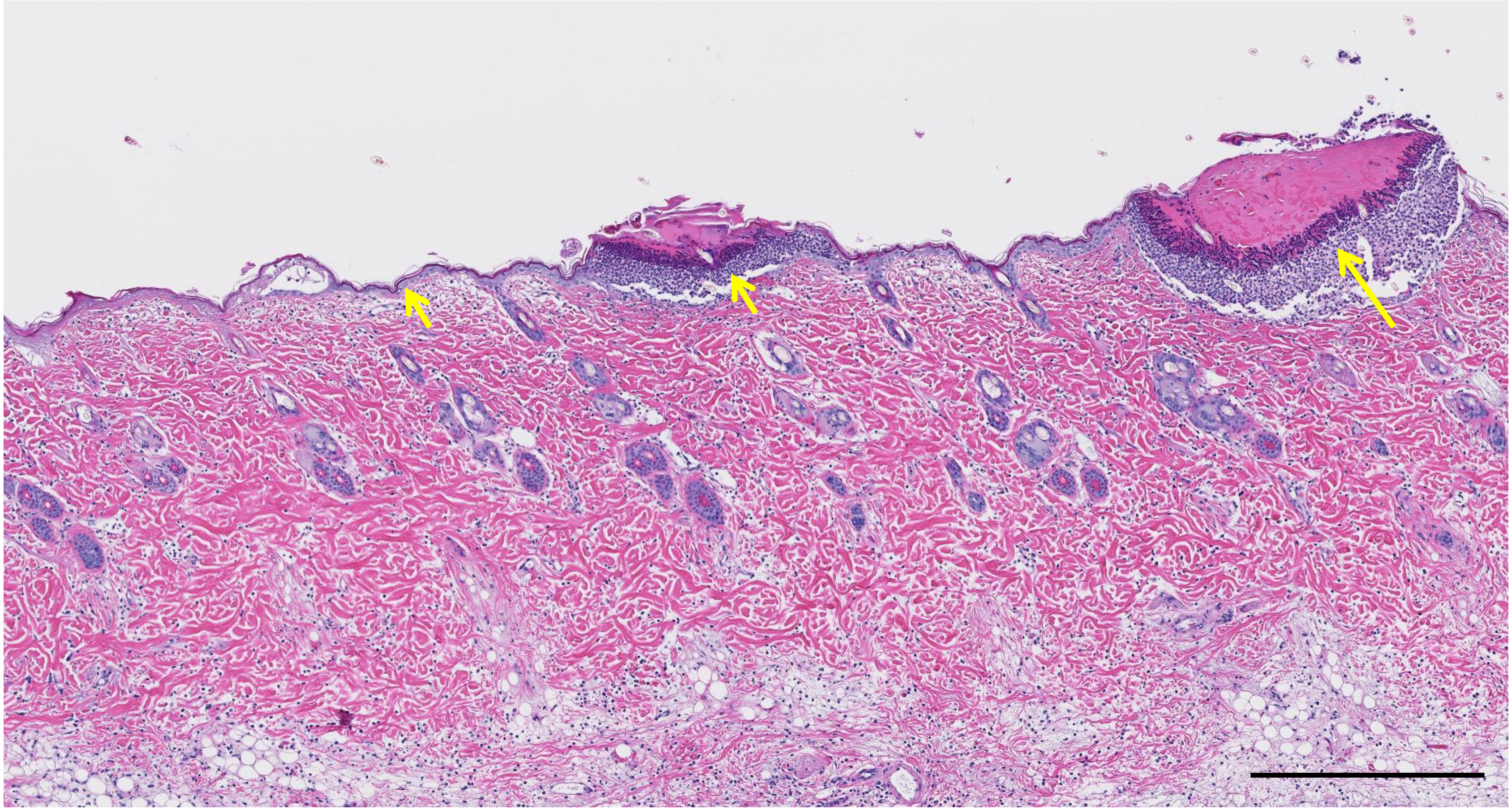

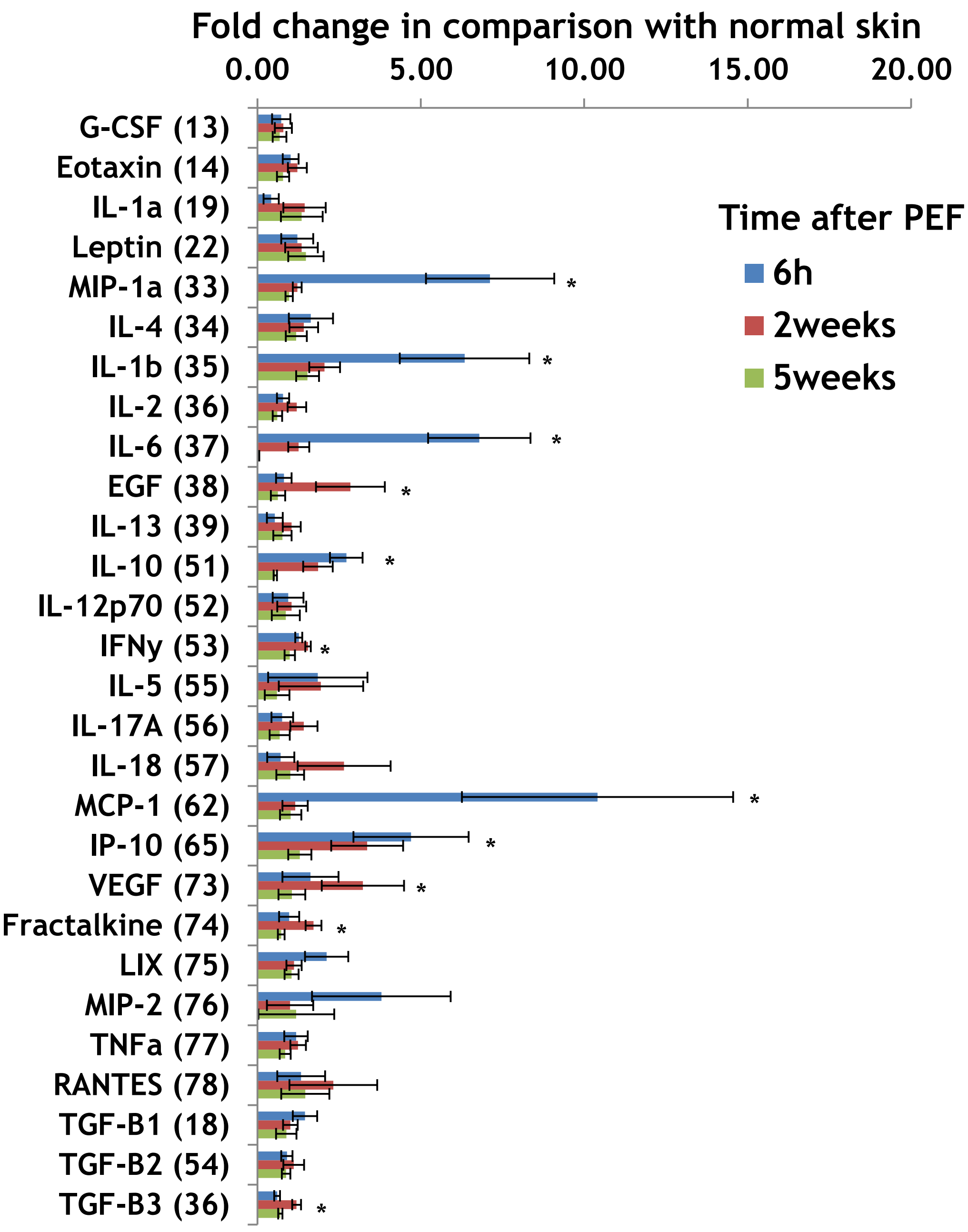

Time after PEF

	6h	2weeks	5weeks
G-CSF (13)	0.73	0.80	0.68
Eotaxin (14)	1.02	1.22	0.79
IL-1a (19)	0.43	1.45	1.36
Leptin (22)	1.22	1.35	1.49
MIP-1a (33)	7.12	1.22	0.97
IL-4 (34)	1.64	1.42	1.19
IL-1b (35)	6.34	2.06	1.54
IL-2 (36)	0.79	1.21	0.61
IL-6 (37)	6.79	1.26	0.04
EGF (38)	0.81	2.85	0.63
IL-13 (39)	0.54	1.05	0.77
IL-10 (51)	2.72	1.85	0.54
IL-12p70 (52)	0.94	1.05	0.87
IFNy (53)	1.27	1.55	0.99
IL-5 (55)	1.85	1.95	0.60
IL-17A (56)	0.76	1.43	0.69
IL-18 (57)	0.72	2.66	1.01
MCP-1 (62)	10.41	1.16	1.02
IP-10 (65)	4.70	3.36	1.30
VEGF (73)	1.62	3.23	1.06
Fractalkine (74)	0.97	1.72	0.73
LIX (75)	2.12	1.13	1.04
MIP-2 (76)	3.80	1.00	1.19
TNFa (77)	1.18	1.25	0.85
RANTES (78)	1.34	2.32	1.47
TGF-B1 (18)	1.46	1.01	0.89
TGF-B2 (54)	0.90	1.11	0.88
TGF-B3 (36)	0.61	1.20	0.70

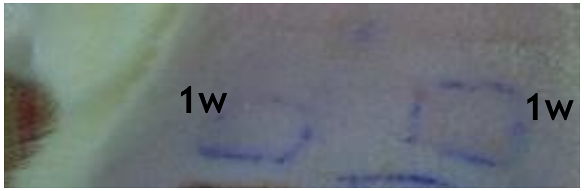
1w
1w
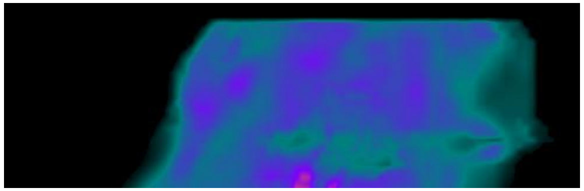

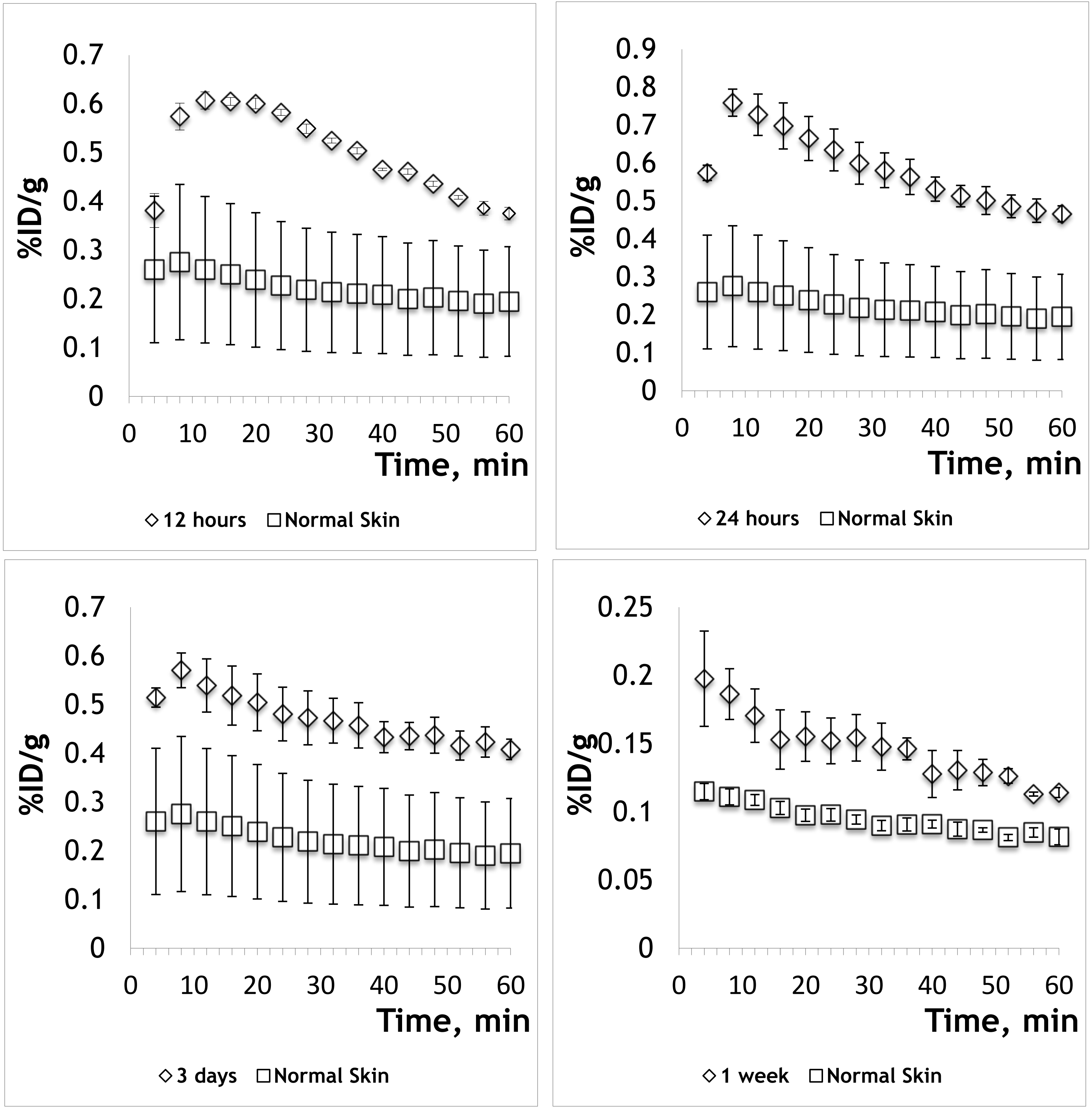
%ID/g
Time, min
◇12 hours □Normal Skin
◇24 hours □Normal Skin
◇3 days □Normal Skin
◇1 week □Normal Skin
0
0.1
0.2
0.3
0.4
0.5
0.6
0.7
0.8
0.9
0.05
0.15
0.25
10
20
30
40
50
60
